# Silencing circATXN1 in Aging Nucleus Pulposus Cell Alleviates Intervertebral Disc Degeneration via Correcting Progerin Mislocalization

**DOI:** 10.34133/research.0336

**Published:** 2024-03-23

**Authors:** Chao Yu, Jing Zhao, Feng Cheng, Jiangjie Chen, Jinyang Chen, Haibin Xu, Kesi Shi, Kaishun Xia, Siwen Ding, Kanbin Wang, Ronghao Wang, Yazhou Chen, Yi Li, Hao Li, Qixin Chen, Xiaohua Yu, Fangwei Shao, Chengzhen Liang, Fangcai Li

**Affiliations:** ^1^ Department of Orthopedics, 2nd Affiliated Hospital, School of Medicine, Zhejiang University, Hangzhou 310009, Zhejiang, PR China.; ^2^ Orthopedics Research Institute of Zhejiang University, Zhejiang University, Hangzhou 310009, Zhejiang, PR China.; ^3^ Key Laboratory of Motor System Disease Research and Precision Therapy of Zhejiang Province, Hangzhou 310009, Zhejiang, PR China.; ^4^ Clinical Research Center of Motor System Disease of Zhejiang Province, Hangzhou 310009, Zhejiang, PR China.; ^5^ Department of Chemistry, Zhejiang University, Hangzhou 310009, Zhejiang, PR China.; ^6^ Westlake Street Community Health Service Center, Hangzhou 310009, Zhejiang, PR China.; ^7^ Zhejiang University-University of Illinois at Urbana-Champaign Institute, Zhejiang University, Haining 314400, Zhejiang, PR China.; ^8^ Biomedical and Health Translational Research Centre, Zhejiang University, Haining 314400, Zhejiang, PR China.

## Abstract

Circular RNAs (circRNAs) play a critical regulatory role in degenerative diseases; however, their functions and therapeutic applications in intervertebral disc degeneration (IVDD) have not been explored. Here, we identified that a novel circATXN1 highly accumulates in aging nucleus pulposus cells (NPCs) accountable for IVDD. CircATXN1 accelerates cellular senescence, disrupts extracellular matrix organization, and inhibits mitochondrial respiration. Mechanistically, circATXN1, regulated by heterogeneous nuclear ribonucleoprotein A2B1-mediated splicing circularization, promotes progerin translocation from the cell nucleus to the cytoplasm and inhibits the expression of insulin-like growth factor 1 receptor (IGF-1R). To demonstrate the therapeutic potential of circATXN1, siRNA targeting the backsplice junction of circATNX1 was screened and delivered by tetrahedral framework nucleic acids (tFNAs) due to their unique compositional and tetrahedral structural features. Our siRNA delivery system demonstrates superior abilities to transfect aging cells, clear intracellular ROS, and enhanced biological safety. Using siRNA–tFNAs to silence circATXN1, aging NPCs exhibit reduced mislocalization of progerin in the cytoplasm and up-regulation of IGF-1R, thereby demonstrating a rejuvenated cellular phenotype and improved mitochondrial function. In vivo, administering an aging cell-adapted siRNA nucleic acid framework delivery system to progerin pathologically expressed premature aging mice (zmpste24^−/−^) can ameliorate the cellular matrix in the nucleus pulposus tissue, effectively delaying IVDD. This study not only identified circATXN1 functioning as a cell senescence promoter in IVDD for the first time, but also successfully demonstrated its therapeutic potential via a tFNA-based siRNA delivery strategy.

## Introduction

Circular RNAs (circRNAs) constitute a category of inherent single-stranded RNAs with a closed circular structure created through an atypical splicing process termed backsplicing [1]. CircRNAs include exons, introns, or both. Compared to linear RNAs, circRNAs possess an extended half-life and display resistance to exonuclease degradation [2]. CircRNAs are found across various tissues and cell types where they perform crucial functions in many biological activities, including development, the preservation of stem cells, and the process of aging [3,4]. Recently, there has been an increasing emphasis on using circRNAs as therapeutic targets to treat age-related diseases [5]. For example, circHIPK3 serves as a scaffold for recruiting ubiquitin ligases, facilitating the degradation of HuR and thus preventing cardiac senescence [6]. Circ-Foxo3 interacts with ID-1, E2F1, FAK, and HIF1α, sequestering them in the cytoplasm and thereby preventing them from performing their antisenescent and antistress functions [7].

Intervertebral disc degeneration (IVDD) is an age-related disease that importantlyimpacts quality of life and is a leading cause of disability [8–10]. Cellular senescence serves as a fundamental mechanism underlying IVDD and is primarily characterized by irreversible growth arrest in nucleus pulposus cells (NPCs) [11,12], ultimately leading to reduced intervertebral height, endplate sclerosis, and depletion of the extracellular matrix (ECM) [13]. Aging NPCs exhibit cell cycle arrest, altered metabolic states, and changes in the expression of cytokines and degradation enzymes. The excessive accumulation of aging NPCs exacerbates the progression of IVDD. However, the majority of related research on IVDD has focused on aspects such as cellular autophagy and apoptosis [14,15]. Although it has been demonstrated that heightened cell apoptosis diminishes the number of disc cells and the amount of ECM, aging NPCs also accumulate in the degenerative intervertebral discs of humans, cattle, and rats [11]. Therefore, the relationship between circRNAs and NPC aging remains to be further explored.

RNA interference (RNAi) is widely utilized in the field of cellular aging research and involves the use of short RNA fragments to suppress gene expression [16,17]. The short RNA segment, widely known as short interfering RNA (siRNA), functions by interrupting gene expression through sequence-specific degradation and the subsequent inhibition of translation in complementary RNAs [18]. Concurrently, the use of biocompatible molecular carrier systems is imperative to overcome the inherent biostability challenges associated with naked siRNA, thereby augmenting the efficiency of RNAi treatment [19]. However, safety concerns and dose-limiting toxicities of commonly used transfection vectors have been identified as the reasons for the current failure to meet the rigorous requirements for clinical applications [20–22]. For example, cationic carriers such as liposomes have been reported to accumulate extensively in the lungs shortly after administration, prompting concerns regarding toxicity [23]. As aging NPCs exhibit reduced metabolic activity, there is currently demand for a safe and efficient transfection reagent.

Recently, tetrahedral framework nucleic acids (tFNAs) have emerged as novel noncationic carriers for drug delivery [24]. Compared with cationic transfection reagents, tFNAs present advantages such as excellent biocompatibility and biodegradability, unparalleled programmability for siRNA delivery [25], natural ability to scavenge reactive oxygen species (ROS) [26], and ease of cellular internalization [27]. tFNAs are formed through the self-assembly of 4 highly specific and programmable DNA single chains (single-stranded DNAs [ssDNAs]). The design of ssDNA enables precise complementary base pairing and sequence customization. In contrast to ssDNA, tFNAs can penetrate mammalian cells without the need for lipofection and maintain structural integrity within the cytoplasm for a minimum of 48 h. These characteristics are vital for their utilization in drug delivery and for regulating their functionality within the cell [28]. As siRNAs used for silencing circRNAs need to possess characteristics that match the backsplice junction sequence, tFNAs can be designed with different complementary strands based on the principles of base-pairing complementarity. Therefore, tFNAs represent a suitable siRNA transfection carrier for aging NPCs.

Herein, by RNA sequencing, we discovered that circATXN1 (circBase ID: has_circ_0007909), a circRNA enriched in aging hNPCs, was up-regulated in aging human nucleus pulposus cells (hNPCs). CircATXN1 is located primarily within the nucleus and around the perinuclear region of hNPCs. The formation of circATXN1 is regulated by heterogeneous nuclear ribonucleoprotein A2B1 (HNRNPA2B1), which recognizes m^6^A sites in the intron 5 region of pre-ATXN. CircATXN1 accelerates cellular senescence, disrupts ECM organization, and inhibits mitochondrial respiration by promoting progerin translocation from the cell nucleus to the cytoplasm and restoring insulin-like growth factor 1 receptor (IGF-1R) expression [29]. To demonstrate the therapeutic effect of circATXN1 in IVDD, we used tFNAs to achieve high siRNA loading and enhance drug endocytosis to substantially suppress the regulatory functions of circATXN1. We constructed a DNA nanogel (SDTET) using tFNAs consisting of 4 DNA strands as well as siRNA as the linker. This system offers excellent biocompatibility, unmatched programmability, a natural ability to scavenge ROS, and efficient gene silencing capability. Mice deficient in Zmpste24 fail to properly cleave prelamin A and exhibit an aging phenotype characterized by severe growth retardation, osteoporosis, IVDD, and a markedly shortened lifespan [30,31]. The SDTET nanogel significantly restored the structure and function of intervertebral discs in Zmpste24^−/−^ mice. Overall, the use of circATXN1 as a therapeutic target and the development of safe and effective SDTET nanogels have propelled further advancements in gene therapy for IVDD.

## Results

### CircATXN1 is up-regulated in aging NPCs and tissue

**Fig. 1.**
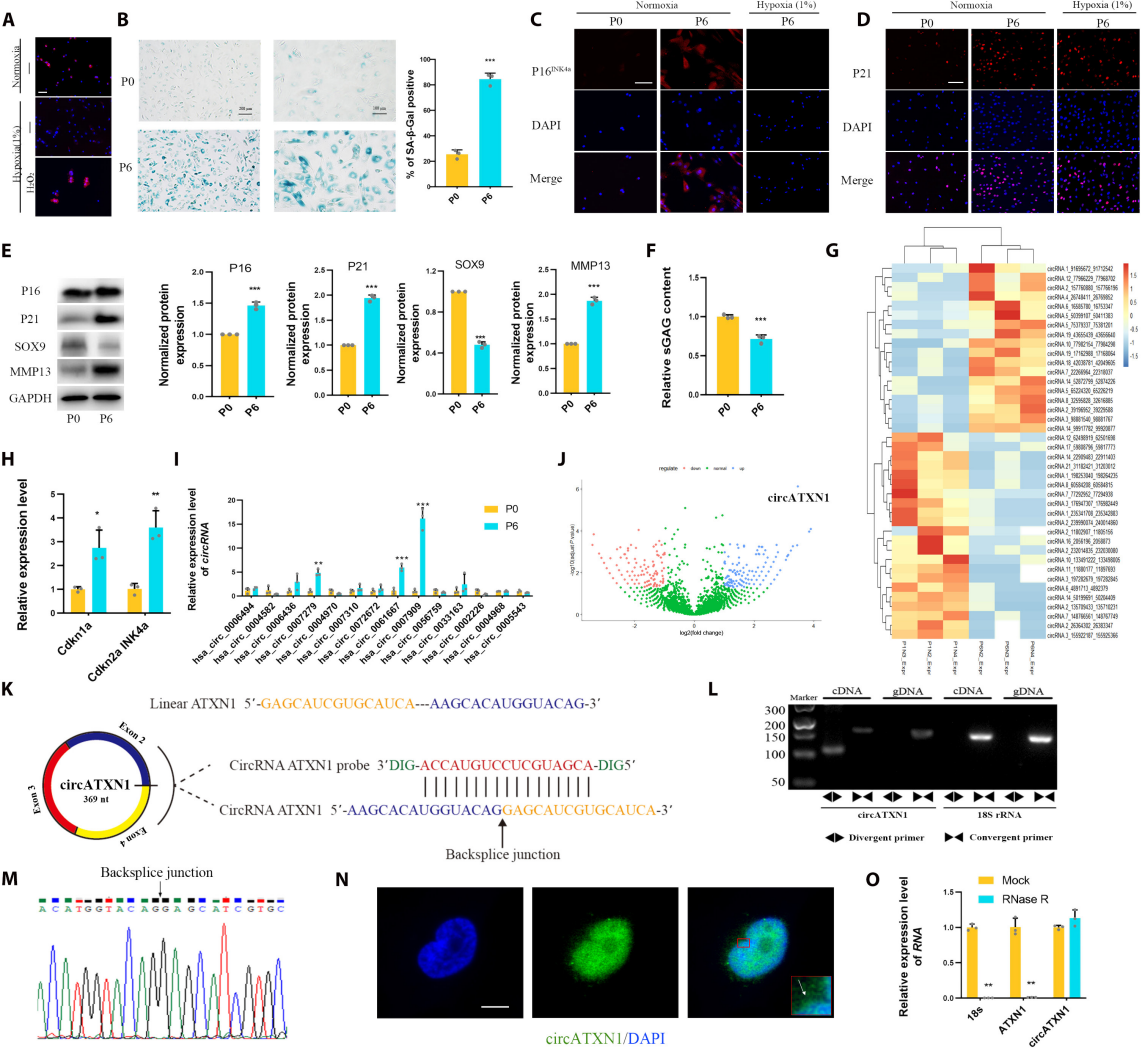
CircATXN1 is up-regulated in aging hNPCs. (A) Immunofluorescence staining of DCFH in primary hNPCs cultured under normoxia (20% O_2_) or hypoxia (1% O_2_) in the absence or presence of H_2_O_2_ (200 μM). Scale bar, 100 μm. (B) hNPCs were exposed to hypoxia at passage 0 (P0) and underwent 6 additional passages under these conditions (P6). SA-β-Gal staining of P0 and P6 hNPCs (*n* = 5). (C and D) Immunofluorescence staining of (C) P16^INK4a^ and (D) P21 in P0 hNPCs cultured under hypoxia or P6 hNPCs cultured under normoxia or hypoxia. DAPI, 4′,6-diamidino2-phenylindole. Scale bar, 100 μm. (E) Semiquantitative analysis of P16, P21, SOX9, and MMP13 protein expression in P0 and P6 hNPCs detected via Western blotting. (F) The release of sGAG from P0 and P6 hNPCs. (G) Heatmap of the circRNA sequencing results. (H) Relative cyclin-dependent kinase (CDK) inhibitor expression in P0 and P6 hNPCs (*n* = 3 biological replicates). (I) Relative expression of circRNAs in P0 and P6 hNPCs. (J) Volcano plot of the circRNA sequencing results. (K) The reverse splicing site of circATXN1 and its corresponding probe sequence. (L) CircATXN1, along with 18S, was amplified from cDNA or gDNA from aging hNPCs via PCR assays with divergent and convergent primers, respectively (*n* = 3 independent experiments). (M) Sanger sequencing of the circATXN1 backsplice junction. (N) RNA in situ hybridization against circATXN1 revealed its subcellular localization. The white arrow indicates the perinuclear localization of circATXN1. Scale bar, 20 μm. (O) RT-qPCR analysis of 18S RNA, ATXN1 mRNA, and circATXN1 levels in aging hNPCs treated with or without RNase R (*n* = 3 biological replicates).

We attempted to identify previously unknown aging-related treatment targets within NPCs that are associated with key IVDD pathological manifestations, such as ECM remodeling and accumulation of oxidative stress. Previous studies have demonstrated that prolonged normoxic culture damages primary cells [32–34]. After comparing the degree of aging in hNPCs cultured under normoxic (20%) and hypoxic (1%) conditions, we subsequently established a stable cellular aging model. Under conditions in which the oxygen concentration is higher than physiological levels, 2′,7′-dichlorofluorescin diacetate (DCFH-DA) probe detection indicated an increase in oxidative stress levels within primary hNPCs (Fig. 1A). Compared to those under low-oxygen conditions (1%), hNPCs exposed to prolonged normoxic conditions (20%) exhibited evident signs of cellular senescence, as indicated by increased senescence-associated β-galactosidase (SA-β-Gal) positivity (Fig. 1B), elevated expression of P16^INK4a^ and P21 (Fig. 1C to E), and up-regulated expression of *Cdkn1a* and *Cdkn2a* (Fig. 1H). Furthermore, the ECM remodeling marker SOX9 was significantly decreased, while matrix metalloproteinase 13 (MMP13) was markedly increased under prolonged normoxic conditions (Fig. 1E). The content of sulfated glycosaminoglycans (sGAG) also decreased under these conditions (Fig. 1F and Fig. S1B and C). Therefore, hNPCs cultured under long-term normoxic conditions represent a stable population of aging cells.

**Fig. 2.**
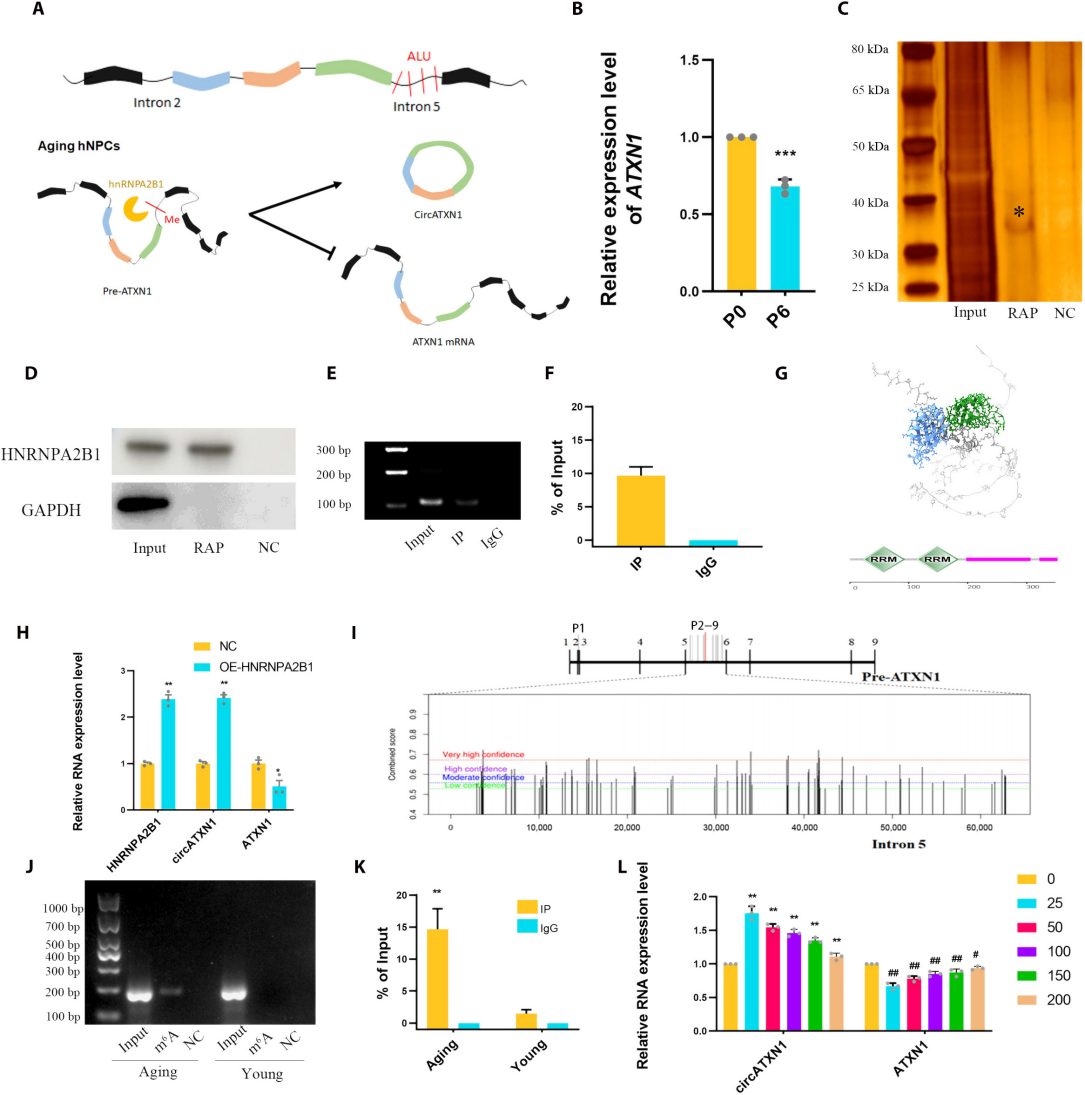
HNRNPA2B1 regulates the splicing of circATXN1 and ATXN1 mRNA in hNPCs. (A) Schematic illustration of the CircATXN1 splicing process. The *Alu* sites are not involved in the mechanism regulating circATXN1 formation. HNRNPA2B1 facilitates the formation of circATXN1 and reduces the generation of ATXN1 mRNA in aging hNPCs. (B) RT-qPCR analysis of ATXN1 mRNA expression in young (P0) and aged (P6) hNPCs. (C) Silver staining of purified interacting proteins in the pre-ATXN1 RAP experiment. NC, scramble control group; RAP, protein group obtained by RNA antisense purification; Input, total protein group. (D) Qualitative analysis of HNRNPA2B1 protein expression in RAP products with different probes was performed via Western blotting. (E and F) HNRNPA2B1 RIP-PCR and RIP-qPCR results for circATXN1 in aging hNPCs. (G) HNRNPA2B1 protein domain analysis (http://smart.embl-heidelberg.de). RRM indicates the 2 functional domains in the HNRNPA2B1 protein secondary structure. (H) RT-qPCR analysis of HNRNPA2B1, circATXN1, and ATXN1 mRNA levels in young hNPCs with and without OE-HNRNPA2B1 treatment (*n* = 3 biological replicates). (I) Schematic representation of the m^6^A site in pre-ATXN1. "P1–9" refers to primers 1 through 9. (J and K) Results of MeRIP-PCR and MeRIP-qPCR analysis of m^6^A in young and aging hNPCs. (L) RT-qPCR analysis of the circATXN1 and ATXN1 mRNA levels in aging hNPCs treated with different concentrations (nM) of STM2457 (*n* = 3 biological replicates).

CircRNAs play essential roles in the aging process. We conducted a comprehensive circRNA expression profile analysis on the aforementioned primary hNPCs cultured under hypoxic conditions and aging hNPCs cultured under normoxic conditions (Fig. S2A). The age, height, weight, and body mass index (BMI) of the patients who donated cells are listed in Table S1. A total of 1,451 unique circRNAs were identified (criteria for selection: log2-fold change greater than 2 or less than −2, with a *P* value of less than 0.05). The source data can be found in the provided Source Data File. These circRNAs are primarily composed of exons and are distributed across various types of chromosomes (Fig. S2B and C). The observed alterations in circRNA levels were validated through reverse transcription-quantitative PCR (RT-qPCR) analysis of the 14 most significantly up-regulated circRNAs (Fig. 1I). For further exploration, we selected the annotated circRNA hsa_circ_0007909, created by the cyclization of exons 3 to 5 of pre-ATXN1, for the following reasons: (a) CircATXN1 was among the most abundant circRNAs in terms of transcripts per kilobase million in our RNA-seq data, particularly among those that were differentially expressed (Fig. 1G and J). (b) CircATXN1 was enriched in the nucleus pulposus tissues of IVDD patients (Fig. S1E) and was involved in regulating sGAG secretion in hNPCs (Fig. S1D). (c) Compared to that in young hNPCs (P0), circATXN1 in aging hNPCs (P6) increased, while ATXN1 mRNA expression decreased (Fig. 1I and Fig. 2B), consistent with previous findings [35]. This evidence indicates that circATXN1 promotes aging in hNPCs.

### CircATXN1 is identified and characterized in hNPCs

Human circATXN1 originates from exons 3 to 5 (chr6: 16586010–16753578) of the ATXN1 gene (Gene ID: 6310; ENSG00000124788), spanning a total length of 369 nucleotides (Fig. 1K). The sequence corresponds to the annotation available in the circBase database (http://www.circbase.org). RNA in situ hybridization demonstrated that circATXN1 was primarily localized within the nucleus and around the perinuclear region in young hNPCs (Fig. 1N). Primers, including both divergent and convergent primers, were designed for circATXN1 as well as its linear transcripts. Amplification and analysis of cDNA and genomic DNA (obtained from aging hNPCs) were performed using agarose gel electrophoresis. The presence of the backsplice junction was verified through Sanger sequencing (Fig. 1L and M). We then examined the stability of circATXN1. After treatment with RNase R, compared with 18S and ATXN1 mRNAs, circATXN1 exhibited increased resistance to RNase R, indicating its distinctive circular feature (Fig. 1O).

### The RNA-binding protein HNRNPA2B1 regulates the formation of circATXN1

Next, we investigated the mechanism of action of circATXN1. Previous studies have indicated that the formation of circRNAs relies primarily on cis elements and RNA-binding proteins (RBPs) [18]. In humans, *Alu* elements are abundantly expressed and serve as regulatory factors for the formation of the majority of circRNAs [18,36]. Hence, we analyzed *Alu* sites within the intronic flanking sequences (intron 2 and intron 5) of circATXN1. We found no *Alu* sites in intron 2. Additionally, through Sanger sequencing, no mutations were observed in the *Alu* sites within intron 5 (Fig. S3A to K). Therefore, we speculate that RNA-binding proteins, rather than cis elements, may regulate the splicing of circATXN1 (Fig. 2A). RT-qPCR analysis revealed an increase in circATXN1 in aging cells (Fig. 1I), while ATXN1 mRNA was down-regulated (Fig. 2B). We designed RNA antisense purification (RAP) probes for pre-ATXN1 and executed RAP assays to uncover proteins that bind to pre-ATXN1. Silver staining revealed a variety of proteins that interact with pre-ATXN1. Specifically, in the molecular weight range of 30 to 40 kDa, uniquely purified proteins were clearly detected (Fig. 2C). The purified proteins were identified using mass spectrometry (MS), and subsequent analysis was carried out using Gene Ontology (GO) analysis (http://www.geneontology.org). MS verified the presence of the HNRNPA2B1 protein (Fig. S3L). GO analysis of the MS data also demonstrated that the pre-ATXN1 binding proteins play an important role in RNA splicing regulation (Fig. S4A).

In light of the results obtained from MS and previous research on RNA-binding proteins [37,38], we substantiated HNRNPA2B1 as a pivotal regulator of circATXN1. Western blot analysis of RAP-purified proteins provided evidence of a direct interaction between HNRNPA2B1 and pre-ATXN1 (Fig. 2D). Among the RNA immunoprecipitation (RIP) products of aging hNPCs, circATXN1 was detected through RIP-PCR and RIP-qPCR analysis (Fig. 2E and F). This evidence further bolsters our aforementioned hypothesis. Structural analysis of HNRNPA2B1 (http://smart.embl-heidelberg.de) revealed the presence of 2 RMs (Fig. 2G). The construction of overexpression plasmids for the treatment of young hNPCs (P0) increased circATXN1 expression and decreased ATXN1 mRNA expression (Fig. 2H). This phenomenon aligns with the observations within aging hNPCs. Therefore, HNRNPA2B1 can regulate the splicing of pre-ATXN1, subsequently influencing the expression of both circATXN1 and ATXN1 mRNA.

In this investigation, we further elucidated the principles governing the modulation of pre-ATXN1 splicing by HNRNPA2B1. HNRNPA2B1 functions as a nuclear reader of the m^6^A mark and mediates alternative splicing [39]. Moreover, the splicing of RNA is also subject to regulation by m^6^A [40]. The potential m^6^A sites within pre-ATXN1 were analyzed (http://www.cuilab.cn/sramp/). We identified numerous m^6^A sites within introns 2/5 of pre-ATXN1 and designed primers (p1 to p9) based on the most likely sites among them (Fig. 2I and Table S3). Based on the findings from MeRIP-PCR and MeRIP-qPCR analyses, we observed a significant increase in the m^6^A level at a specific site (sequence context: CUAGA AUCAC AGACC AUUAG GG ACU GUAGA GAUCU AACAU GCCCA) in aging cells compared to their younger counterparts. As previously reported, HNRNPA2B1 is regulated through METTL3-dependent m^6^A RNA methylation [41]. We employed the METTL3 inhibitor STM2457 to validate the regulatory function of METTL3/HNRNPA2B1/pre-ATXN1 [42]. With increasing concentrations of STM2457, there was a gradual decrease in the level of circATXN1 in aged hNPCs, while the change in the ATXN1 mRNA showed the opposite trend (Fig. 2L). Overall, these findings suggest that the differential expression of ATXN1 mRNA and circATXN1 in aging hNPCs could be the result of coregulation by HNRNPA2B1 and pre-ATXN1 methylation.

### CircATXN1 promotes the senescence of hNPCs by inducing the mislocalization of progerin

**Fig. 3.**
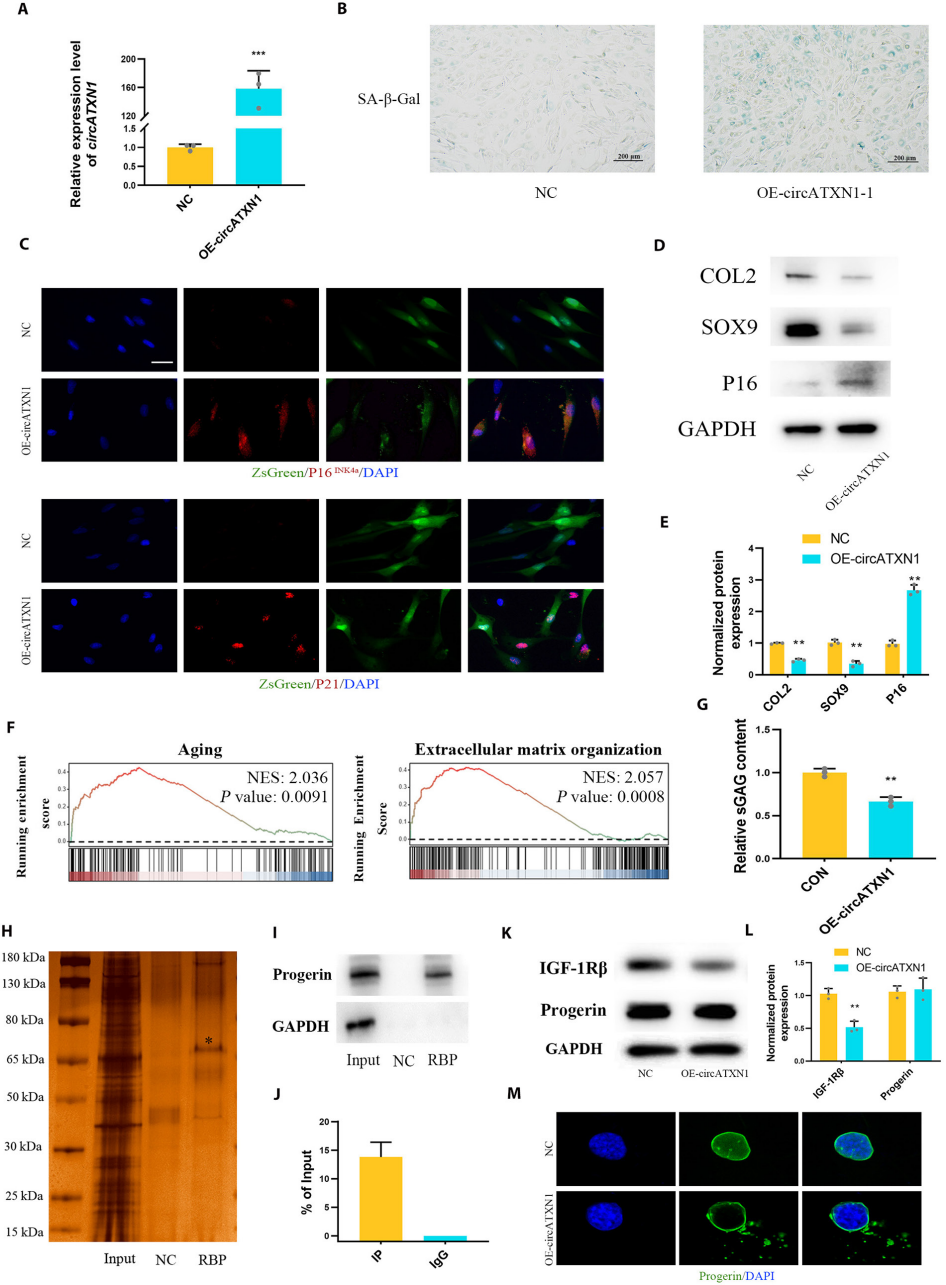
Characteristics and biological functions of circATXN1. (A) RT-qPCR analysis of circATXN1 levels in young hNPCs (P0) with and without OE-circATXN1 treatment (*n* = 3 biological replicates). (B) SA-β-Gal staining of young hNPCs with and without OE-circATXN1 treatment. (C) Immunofluorescence staining of P16^INK4a^ and P21 in young hNPCs with and without OE-circATXN1 treatment. Scale bar, 50 μm. (D) Western blotting showing COL2, SOX9, and P16 protein expression in young hNPCs with and without OE-circATXN1 treatment. (E) Semiquantitative results from Western blotting (*n* = 3 biological replicates). (F) GSEA of young hNircXN1-overexpressing cells treated with or without OE-circATXN1 for 48 h. NES, normalized enrichment score. (G) Relative sGAG content of young hNPCs with and without OE-circATXN1 treatment (*n* = 3 biological replicates). (H) Silver staining of purified interacting proteins from the CircATXN1 RNA pull-down experiment. NC, scramble control group; RBP, protein group obtained by circATXN1 probe purification; Input, total protein group. (I) Western blot analysis of the protein expression of progerin in RBP products. (J) Progerin RIP-qPCR results for circATXN1 and GAPDH in aging hNPCs. (K and L) Western blot analysis of IGF-1Rβ and progerin protein expression. (M) Immunofluorescence staining showing the mislocalization of progerin in the cytoplasm. Scale bar, 10 μm.

To investigate the relationship between circATXN1 and hNPC aging, we constructed an overexpression plasmid and used a lentivirus to overexpress circATXN1 (OE-circATXN1). At 48 h posttreatment, the expression level of circATXN1 in the overexpression group was nearly 160 times greater than that in the nontransfected group (Fig. 3A). This result indicates the effectiveness of the overexpression. OE-circATXN1 significantly promoted the aging phenotype in young hNPCs, as indicated by an increase in the percentage of SA-β-gal-positive cells (Fig. 3B) and elevated protein expression of p16^INK4a^ and p21. Due to the inclusion of the ZsGreen sequence in OE-circATXN1, we observed that ZsGreen-positive cells following OE-circATXN1 treatment exhibited elevated expression of both P16^INK4a^ and P21 (Fig. 3C). Moreover, OE-circATXN1 also influenced ECM remodeling in young hNPCs, as evidenced by the down-regulation of COL2 and SOX9 protein expression (Fig. 3D). The semiquantitative results from Western blotting demonstrated the same findings (Fig. 3E). Dimethyl methylene blue (DMMB) staining indicated a significant decrease in the cellular sGAG concentration after circATXN1 was overexpressed (Fig. 3G). To comprehensively understand the biological effects of OE-circATXN1, we conducted transcriptome sequencing of both hNPCs and hNPCs overexpressing circATXN1. The results of gene set enrichment analysis (GSEA) revealed enrichment in the aging (NSE: 2.036; *P* value: 0.0091) and ECM organization (NSE: 2.057; *P* value: 0.0008) pathways (Fig. 3F), consistent with the aforementioned results. Therefore, in conjunction with the evidence presented, these findings suggest that circATXN1 is crucially involved in the process of hNPC aging and has an impact on the stability of the ECM in these cells.

To elucidate the precise mechanism of aging in NPCs, we explored the specific biological functions of circATXN1 in NPCs. CircRNAs can potentially lead to various biological effects, including acting as miRNA sponges, enhancing protein function, serving as scaffolds to facilitate complex formation between specific enzymes and substrates, and recruiting proteins to specific locations [1]. We initially conducted RIP experiments using the Argonaute2 (AGO2) protein. After removing the RNA from the purified solution, we conducted RT-qPCR analysis and did not detect the presence of circATXN1. These findings suggested that circATXN1 might not exert its effects by functioning as a miRNA sponge.

Furthermore, we designed circATXN1-specific probes and conducted RNA pull-down experiments in an attempt to investigate the proteins that interact with circATXN1. Silver staining revealed numerous proteins that interact with circATXN1. Within the molecular weight range of 65 to 80 kDa, there was a clear presence of specific purified proteins (Fig. 3H). In light of the MS results, we confirmed that progerin is a binding protein of circATXN1 (Fig. S6E). Several studies have suggested that progerin plays a regulatory role in physiological aging [29,31,43]. Additionally, the aging process and mitochondrial dysfunction in hNPCs are also correlated with progerin [43]. Western blot analysis of RBP-purified proteins also provided evidence of a direct interaction between progerin and circATXN1 (Fig. 3I). Among the RIP products of the aging hNPCs, circATXN1 was detected through RIP-qPCR analysis (Fig. 3J). These findings demonstrated the direct interaction between progerin and circATXN1.

The main reasons why the progerin protein induces cellular aging include genomic instability [44], nuclear blebbing [45], and mislocalization in the cytoplasm [29]. WB analysis of the biological characteristics of circATXN1 revealed that the protein expression of progerin was unchanged in hNPCs after circATXN1 overexpression (Fig. 3K). Furthermore, mislocalization of the progerin protein led to down-regulation of IGF-1Rβ [29], and overexpression of circATXN1 promoted this phenomenon in young hNPCs (Fig. 3K and L). Therefore, we propose that circATXN1 is involved in the mislocalization of progerin. Immunofluorescence assays revealed an increase in the extent of progerin mislocalization following the overexpression of circATXN1 (Fig. 3M). In summary, circATXN1 promotes the cytoplasmic localization of progerin, which affects the IGF-1Rβ signaling pathway, thereby promoting the aging process in hNPCs.

### Programmed assembly and characterization of siRNA–DNA tetrahedrons

**Fig. 4.**
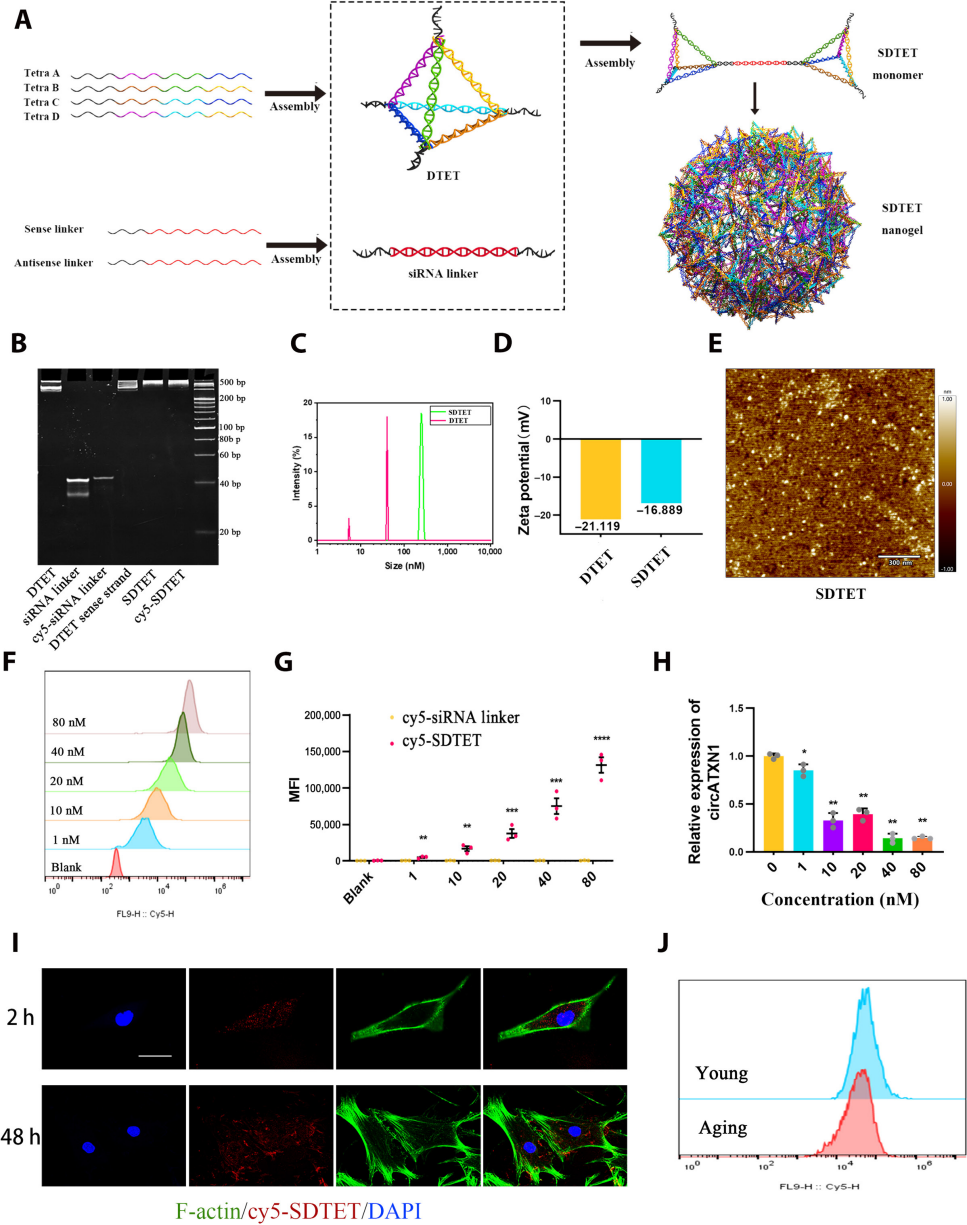
Cellular uptake behaviors of the SDTET nanogel. (A) Schematic diagram of the DNA tetrahedral structure. (B) Polyacrylamide gel electrophoresis (PAGE) analysis. (C) Dynamic light scattering of the DTET and SDTET nanogels. (D) Zeta potential analysis of the SDTET nanogel and DTET. (E) The morphology of the SDTET nanogel revealed by AFM imaging. (F) Flow cytometry analysis of aging hNPCs incubated with the cy5-labeled SDTET nanogel and siRNA linker for 2 h at different concentrations. (G) Quantitative analysis of the MFI via flow cytometry analysis. (H) RT-qPCR analysis of circATXN1 levels in aging hNPCs treated with different concentrations of the SDTET nanogel (*n* = 3 biological replicates). (I) Confocal microscopy images of aging hNPCs after treatment with the cy5-labeled SDTET nanogel for 2 or 48 h. Scale bar, 20 μm. (J) Flow cytometry analysis of aging and young hNPCs incubated with the cy5-labeled SDTET nanogel (40 nM) for 2 h.

RNAi-mediated degradation strategies have been widely employed to interfere with the expression of circRNAs [18]. To reduce the expression of circATXN1, we constructed a DNA nanogel (SDTET) using a DNA tetrahedron (DTET) consisting of 4 DNA strands as well as siRNA as the linker (Fig. 4A). In the current field of circRNA research, lipid-based transfection reagents are predominantly utilized [46,47]. Compared to cationic liposomes, the SDTET nanogel exhibits no significant cytotoxicity, which makes it a suitable transfection system for aging hNPCs. In conclusion, we developed a nanogel based on DTETs that can effectively transport siRNA to aging hNPCs without causing any harm to the cells.

DTET consists of four 69-base DNA strands, each with a 12-base terminal extension that can be used for hybridization with our designed siRNA linker through complementary sticky regions (Table S2, DNA and RNA sequences are included). Next, we combined the assembled DTETs with the siRNA linker at a carefully optimized ratio of 1:1.8 to form a nanosized SDTET nanogel embedded with siRNA. The resulting SDTET nanogel was subsequently examined using agarose gel electrophoresis and native polyacrylamide gel electrophoresis (PAGE) (Fig. 4B and Fig. S5A). A smear was observed in the band of the nanogel, and the SDTET nanogel migrated significantly slower than both the siRNA linkers and the DTET, indicating that the efficient assembly of the siRNA-loaded nanogel was successful. Dynamic light scattering (DLS) and atomic force microscopy (AFM) were employed to examine the morphology and size distribution of the SDTET nanogel. The hydrodynamic diameter of the DTET was approximately 40 nm, and the resulting SDTET nanogel was significantly larger (250 nm), which is suitable for cellular delivery (Fig. 4C). The zeta potentials of the DTET and SDTET nanogels were −21.119 and −16.889 mV, respectively (Fig. 4D). Furthermore, AFM imaging revealed that the assembled SDTET nanogel exhibited a spherical morphology. (Fig. 4E).

### Biocompatibility, internalization, and silencing efficiency of the SDTET nanogel

After successfully preparing the SDTET nanogel, we proceeded to confirm its biocompatibility, effective internalization, and silencing efficiency. Given that previous studies have indicated that cationic nanocarriers can lead to substantial cellular toxicity [48], we focused on the transfection safety of the SDTET nanogel in aging hNPCs compared to that of 1,2-dioleoyl-3-trimethylammonium-propan (DOTAP) liposomes. We treated aged hNPCs with DOTAP liposomes (25 μg/ml) and the SDTET nanogel (25 μg/ml) for 2 h. Cell counting kit-8 (CCK-8) assays indicated that DOTAP liposomes reduced the viability of aging hNPCs (Fig. S5C). Furthermore, hNPCs treated with DOTAP liposomes exhibited alterations in cell morphology and intracellular ROS accumulation (Fig. S5D). The treated cells exhibited swollen cytoplasm and ruptured plasma membranes (Fig. S5E). Nonetheless, hNPCs treated with DTET displayed a rejuvenated cellular morphology, possibly due to the ability of the DTET to cleave ROS.

To investigate the internalization ability of the SDTET nanogel, a siRNA linker was labeled with cyanine 5 (cy5) to track the internalization of the SDTET nanogel inside the hNPCs. Flow cytometry analysis was employed to assess the efficiency of the SDTET nanogel in terms of cy5 uptake by aging hNPCs treated with various concentrations (1, 10, 20, 40, and 80 nM). Specifically, Cy5-labeled siRNA and Cy5-SDTET nanogel were separately coincubated with aging hNPCs for 2 h. The findings from both flow cytometry analysis and the quantitative assessment of the mean fluorescence intensity (MFI) consistently illustrated that the internalization of the SDTET nanogel into cells exhibited a concentration-dependent trend (Fig. 4F and G). At these concentrations, the SDTET nanogel did not exhibit significant cytotoxicity (Fig. S5F).

To evaluate the gene silencing efficiency of the SDTET nanogel upon uptake by aging hNPCs, we treated cells with varying concentrations of the SDTET nanogel. The RT-qPCR results revealed a reduction in circATXN1 expression in aging hNPCs with increasing concentrations of the SDTET nanogel (Fig. 4H). In terms of knockdown efficiency, we opted to use an SDTET nanogel at a concentration of 40 nM for subsequent investigations. This decision is rooted in the fact that at this safe concentration, the knockdown efficiency is relatively high, thereby preventing potential cellular damage that might arise from higher concentration treatments. Confocal microscopy images also indicated the presence of the SDTET nanogel within hNPCs after 2 h of treatment (Fig. 4I). Moreover, even after 48 h, a considerable amount of the SDTET nanogel remained detectable within the hNPCs. Finally, we compared the transfection capacity of the SDTET nanogel between young and aging hNPCs. Flow cytometry analysis revealed that the SDTET nanogel maintained its strong transfection ability in aging cells compared with young cells (Fig. 4J). In summary, the experimental results indicate that the SDTET nanogel has excellent cell-penetrating ability, circATXN1 knockdown capability, and biocompatibility with aging hNPCs.

### SDTET nanogel reverses the aging process in aging hNPCs

**Fig. 5.**
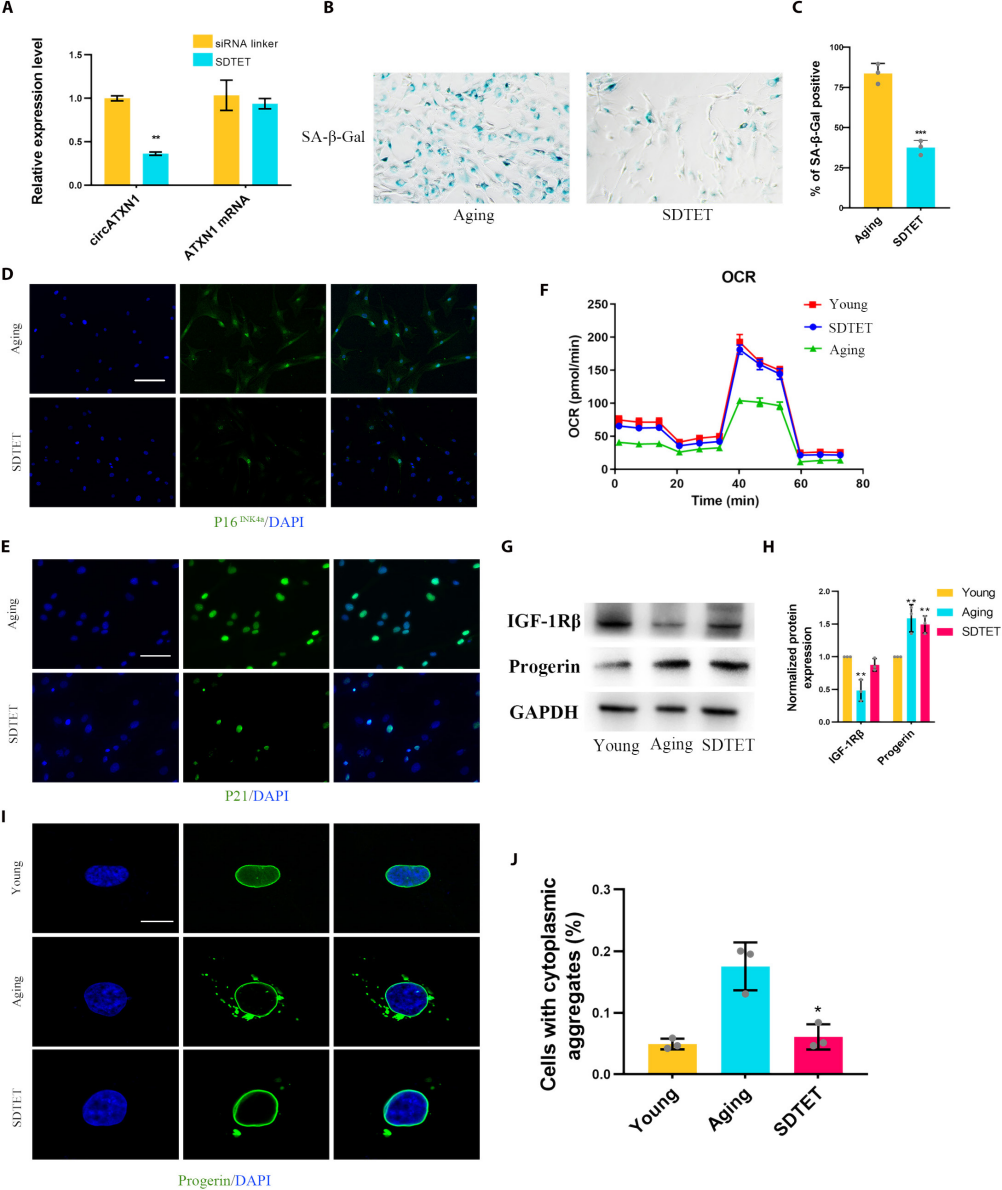
The SDTET nanogel slows the aging process in aging hNPCs. (A) RT-qPCR analysis of circATXN1 levels in aging hNPCs treated with a siRNA linker (si-NC) or the SDTET nanogel (*n* = 3 biological replicates). (B) SA-β-Gal staining of aging hNPCs after treatment with si-NC or the SDTET nanogel. (C) Semiquantitative analysis of SA-β-Gal staining (*n* = 3 biological replicates). (D and E) Immunofluorescence staining of p16^INK4a^ and p21 in aging hNPCs with or without SDTET nanogel treatment. Scale bar, 100 μm. (F) OCR analysis of aging hNPCs with or without the SDTET nanogel treatment (*n* = 6 biological replicates). (G and H) Western blot analysis of IGF-1Rβ and progerin protein expression. (I) Immunofluorescence staining showing the mislocalization of progerin in the cytoplasm. Scale bar, 10 μm. (J) Analysis of the proportion of cells with cytoplasmic mislocalization. Young, P0 hNPC group; Aging, P6 hNPC group; SDTET, P6 hNPC treated with the SDTET nanogel group.

We further investigated the biological effects of the SDTET nanogel on aging hNPCs. Compared with the siRNA linker, the SDTET nanogel significantly reduced the expression of circATXN1 in aging hNPCs without affecting the expression of ATXN1 mRNA (Fig. 5A). Aging hNPCs displayed a rejuvenation phenomenon after SDTET nanogel treatment, which was primarily characterized by a reduction in SA-β-Gal positivity (Fig. 5B and C) and a decrease in the expression of P16^INK4a^ and P21 (Fig. 5D and E and Fig. S6B and C). The oxygen consumption rate (OCR) data indicate that the glycolytic capacity, mitochondrial reserve capacity, maximum aerobic capacity, and non-glucose-dependent respiration of hNPCs decrease as aging progresses. Interestingly, aging hNPCs treated with the SDTET nanogel exhibited a glycolytic capacity resembling that of young cells (Fig. 5F). The sGAG content in aging hNPCs also increased after SDTET nanogel treatment (Fig. S6A). After SDTET treatment, the expression of IGF-1Rβ was restored in aging NPCs, but there was no significant change in the level of progerin itself (Fig. 5G and H and Fig. S6H). Moreover, the number of hNPCs exhibiting cytoplasmic aggregation of progerin decreased after circATXN1 knockdown (Fig. 5I and J and Fig. S6F). Our study demonstrated that the SDTET nanogel possesses a precise and remarkable ability to knock down circATXN1, leading to the rejuvenation of aging hNPCs. In conclusion, the SDTET nanogel exhibits highly efficient circATXN1 silencing without cellular toxicity, making it an excellent innovative vector for rejuvenating aging hNPCs via gene therapy.

### The SDTET nanogel delays IDD in zmpste24^−/−^ mice by silencing circATXN1

First, we detected the conserved mouse circRNA (formed by cyclization of exons 2 to 4 of mouse pre-ATXN1; referred to as ms-circATXN1 in a subsequent study) of circATXN1. Based on the backsplice junction site primer design, we confirmed the presence of ms-circATXN1 in mouse nucleus pulposus cells (mNPCs) through PCR detection (Fig. S7A). With the use of the ms-circATXN1 sequence, we designed new siRNA sequences and loaded them into the DTET vector (Table S2). After SDTET nanogel treatment, the expression of ms-circATXN1 decreased in the aging mNPCs (Fig. S7B). Moreover, aging mNPCs exhibited alleviation of aging phenotypes, as indicated by a decrease in the percentage of SA-β-Gal-positive cells, an increase in the protein expression of p16^Ink4a^, and an increase in the ECM (Fig. S7C and D). These findings demonstrated that ms-circATXN1 exerts biological effects in a manner similar to that of human circATXN1, indicating that it has a conserved homologous function in mice.

**Fig. 6.**
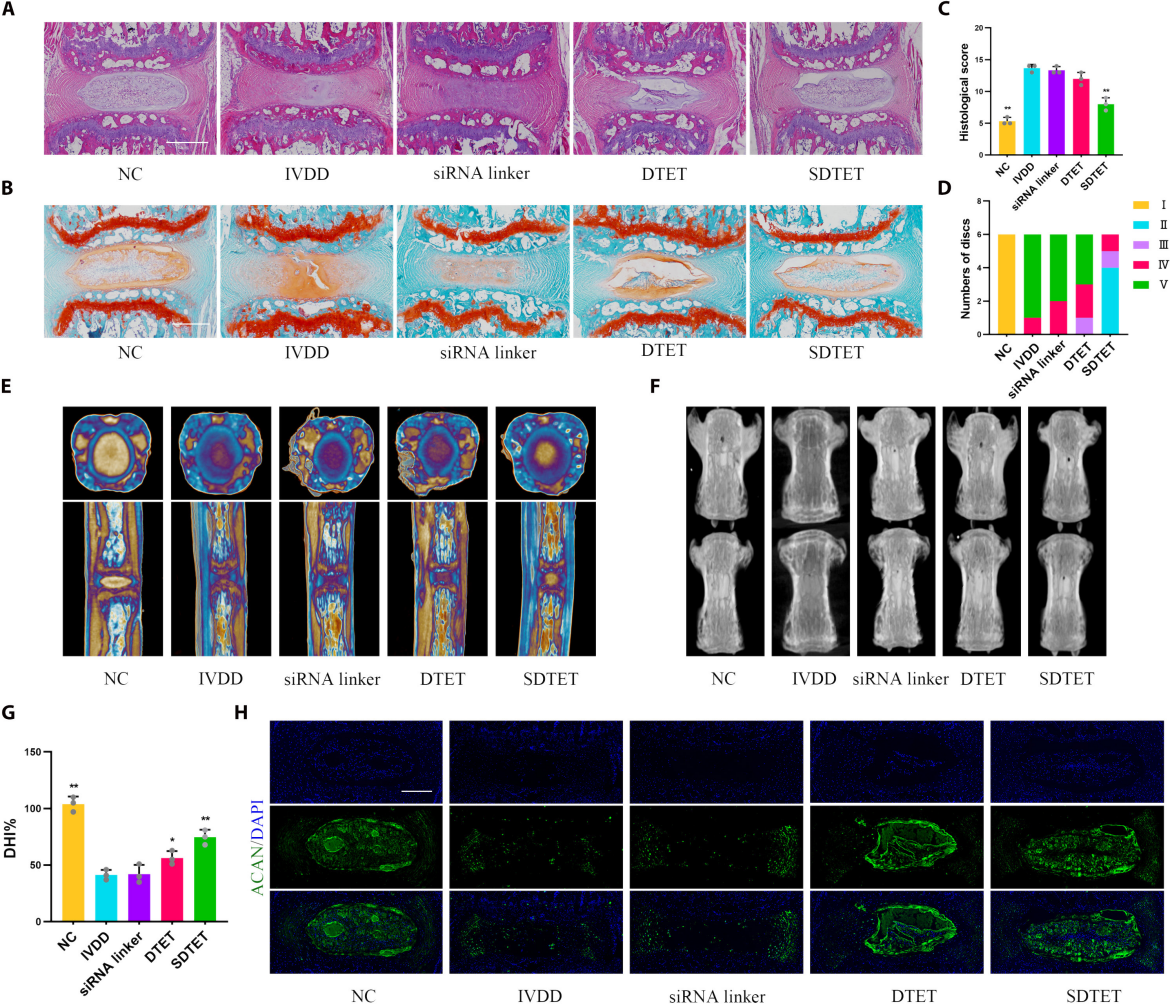
The SDTET nanogel retards the progression of IVDD in zmpste24^−/−^ mice (A) HE staining of nucleus pulposus tissue under different treatment conditions. (B) SO staining of nucleus pulposus tissue under different treatment conditions. Scale bar, 500 μm. (C) The histological score was used to evaluate the tissue structure of the nucleus pulposus (*n* = 3 biological replicates). Scale bar, 500 μm. (D) The Pfirrmann grading system was used to assess the nucleus pulposus structure comprehensively in terms of fibrous ring clarity, signal intensity, and height (*n* = 6 biological replicates). (E and F) Micro-CT analysis of the intervertebral disc. (G) DHI was used to evaluate the disc height in the different groups. (H) Immunofluorescence staining of ACAN revealed the ECM content in various nucleus pulposus tissue compartments. Scale bar, 500 μm. NC, the untreated control group; IVDD, the naturally aged degeneration group; siRNA, the in situ siRNA injection group; DTET, the in situ DTET injection group; SDTET, the in situ SDTET nanogel injection group. *P < 0.05 compared with the IVDD group. **P < 0.01 compared with the IVDD group.

Zmpste24^−/−^ mice exhibit significant mislocalization of progerin within the cell cytoplasm [29]. Knocking out Zmpste24 leads to premature aging and damage to the ECM in intervertebral discs [30]. Therefore, we chose zmpste24^−/−^ mice as an in vivo aging-related IVDD experimental model. From a structural analysis perspective, hematoxylin–eosin (HE) staining and safranin O (SO) staining revealed that the nucleus pulposus tissue displayed a well-preserved tissue structure after treatment with the SDTET nanogel (Fig. 6A and B). The intervertebral disc structure also showed signs of recovery after DTET treatment, primarily attributed to the ROS-scavenging properties of DTET itself [24]. The histological score of the cells in the SDTET nanogel treatment group was significantly lower than that of the other groups (Fig. 6C). We conducted Piffmann grading assessments on the different intervertebral discs under study. The Piffmann grade in the IVDD group was mainly at level V, whereas in the SDTET nanogel group, it was mainly at level II (Fig. 6D). Micro-computed tomography (Micro-CT) analysis also demonstrated that the patients in the SDTET nanogel group exhibited improved intervertebral disc structure and height (Fig. 6E and F). A statistical analysis of DHI revealed that the intervertebral disc height in the SDTET nanogel group was approximately 77% that of the normal group, while that in the other groups was generally less than 50% (Fig. 6G). Immunofluorescence analysis of ACAN-targeting cells indicated that, compared with that in the IVDD group, the ECM content within the nucleus pulposus in the SDTET nanogel treatment group was increased (Fig. 6H). Consequently, zmpste24^−/−^ mice exhibit noticeable degeneration of their intervertebral discs as they age. This degeneration is partly attributed to the mislocalization of progerin. The SDTET nanogel rejuvenates nucleus pulposus tissue/cells by interfering with circATXN1 expression and reducing the mislocalization of progerin.

## Discussion

IVDD is a complex process influenced by both age-related alterations and tissue damage resulting from various stresses. The buildup of cellular senescence is associated with reduced cellular proliferation, compromised self-repair mechanisms, elevated inflammatory reactions, and heightened catabolic metabolism [11]. CircRNAs, a category of endogenous single-stranded closed-loop RNAs, play a role in cellular aging-related biological processes [5]. CircRNAs primarily function as regulators within cells. Currently, most circRNAs primarily function as molecular sponges of miRNAs. CircACVR2A can act as a miR-626 sponge, modulating the expression of the EYA4 gene [49]. CircRNAs can also be translated into peptide segments or proteins. Circ-FBXW7 contains an open reading frame (ORF), which enables internal ribosomes to reach the corresponding site to encode a novel protein (FBXW7 to 185aa) [50]. Furthermore, circRNAs play pivotal roles in the regulation of gene transcription. CIRC EIF3J and CIRC PAIP2 can enhance the expression of their parent genes [51]. CircRNAs can serve as scaffolds, bind to proteins, and facilitate interactions between proteins. CircFOXO3 accelerates cardiac aging by strengthening its associations with the anti-aging protein ID-1, the transcription factor E2F1, and the anti-stress protein FAK [7]. Interestingly, circRNAs can also influence the subcellular localization of proteins. CircAGO2 can interact with HuR and promote its translocation from the nucleus to the cytoplasm [52]. A growing body of evidence indicates that circRNAs are involved in the onset and progression of IVDD [14].

In our research, the role of circATXN1 in promoting aging in hNPCs was first identified. Bioinformatics analysis and RAP experiments revealed that the splicing of circATXN1 is controlled by HNRNPA2B1. Furthermore, this regulatory effect is activated by the m^6^A site in pre-ATXN1 intron 5. Although the expression of ATXN1 mRNA is similarly regulated, the biological effects resulting from its down-regulation in aging hNPCs have not been determined. However, further research is required to explore this phenomenon. Through the construction of a plasmid for circATXN1 overexpression, we observed a distinct aging phenotype in young hNPCs, which included elevated expression of P16^Ink4a^ and p21, increased SA-β-Gal positivity, and remodeling of the ECM. Further investigations revealed that circATXN1 can bind to progerin and promote its translocation from the nucleus to the cytoplasm, consequently enhancing its association with IGF-1Rβ. The down-regulation of IGF-1Rβ leads to cellular aging [29]. These data suggest that down-regulating circATXN1 may be a strategy for rejuvenating hNPCs.

RNAi is extensively employed in the field of circRNA research and involves the use of short RNA fragments to silence gene expression. Despite the existence of numerous siRNA delivery vectors, these carriers still pose significant issues related to cellular toxicity and transfection efficiency [53]. The DTET is a novel highly programmable siRNA delivery vector. The DTET possesses a natural ability to scavenge ROS. It also exhibits cellular uptake and tissue permeability based on its size and geometric shape, along with high programmability rooted in the principles of base pairing complementarity [24].

To reduce the expression of circATXN1, we found that currently widely used cationic transfection reagents, such as Lipofectamine, cannot be used to efficiently transfect aging hNPCs. Lipofectamine exhibits noticeable cationic toxicity in aging hNPCs. Therefore, we designed siRNAs based on the backsplicing junction site of circRNAs and loaded them onto a DNA tetrahedral structure. Flow cytometry analysis indicated that the SDTET nanogel exhibited excellent cellular penetration ability. We also demonstrated for the first time the circRNA silencing ability of the SDTET nanogel. The accumulation of progerin within zmpste24^−/−^ mouse cells leads to aging-related IVDD. Using the zmpste24^−/−^ mouse as an animal model, we observed that treatment with the SDTET nanogel can mitigate the progression of IVDD induced by aging. The partial restoration of nucleus pulposus morphology and function observed in the DTET-treated group may be attributed to DTET itself possessing a certain capacity for ROS clearance. In summary, the novel transfection agent SDTET, a nanogel, has excellent gene silencing capabilities and biocompatibility in aging hNPCs, which are typically considered challenging to transfect.

Our study has several limitations to consider. First, ATXN1 mRNA is also regulated during the pre-ATXN1 splicing process. However, its biological role in aging hNPCs requires further exploration. Second, the specific mechanism by which circATXN1 regulates the mislocalization of progerin to the cytoplasm remains unknown. Future objectives include elucidating the exact binding sites and translocation principles between circATXN1 and progerin. Meanwhile, we will further analyze the specific mechanisms of nuclear cell aging through single-cell sequencing in future studies [54]. Finally, the SDTET nanogel demonstrated efficient and safe transfection capabilities for aging cells. Nevertheless, the structure of the backsplicing junction site poses a risk of off-target effects in siRNA silencing strategies. Therefore, the development of a DTET with enhanced targeting capabilities is a promising research direction.

In conclusion, this study demonstrated that the SDTET nanogel regulates hNPC aging and ECM remodeling by interfering with circATXN1/progerin/IGF-1Rβ signaling. These discoveries contribute to an enhanced understanding of IVDD pathogenesis and could assist in the development of potentially efficacious strategies for treating IVDD.

## Materials and Methods

### Human nucleus tissue and cells

Nucleus pulposus samples from humans were obtained courtesy of the Second Affiliated Hospital, Zhejiang University School of Medicine (Hangzhou, China). These samples were then thoroughly separated and segmented. Subsequently, a 1% type II collagenase solution (Beyotime) was used to digest the tissue suspension for 1 h. The cells were cultivated in Dulbecco’s modified Eagle’s medium (Corning) supplemented with 10% fetal serum albumin (Gibco), 100 U/ml penicillin, and 100 μg/ml streptomycin and were incubated at 37°C in a 5% CO_2_ environment. Primary cells were cultivated in 1% oxygen. During extended cell culture under normoxic conditions, the oxygen concentration was maintained at 20%. Clinicopathological data were acquired from the Second Affiliated Hospital, Zhejiang University School of Medicine. The study included patients who experienced disc injuries due to trauma. Ultimately, the study included 5 young and 5 elderly patients (Table S1). Informed consent was obtained from all participants. The human studies adhered to the Declaration of Helsinki principles and received approval from the Ethics Committees of the Second Affiliated Hospital, Zhejiang University School of Medicine (Hangzhou, China).

### RNA sequencing and analysis

Primary hNPCs were cultivated in low-oxygen environments, whereas hNPCs from older donors were cultured under conditions with normal oxygen levels. Both of these cellular varieties were subsequently advanced to the RNA sequencing stage. This sequencing and subsequent analysis were carried out by Majorbio Company (Shanghai, China). Using an RNA isolation kit (Vazyme), RNA was extracted from more than 200 islets per group. The RNA purity was evaluated with a Nanodrop 2000, and its integrity was assessed using the Agilent 2100 Bioanalyzer System from Agilent Technologies. For the preparation of sequencing libraries, we followed the guidelines provided in the VAHTSTM Total RNA-seq (H/M/R) Library Prep Kit for Illumina. After cluster generation, these libraries were subjected to sequencing on the Illumina HiSeq X10 platform, which resulted in the generation of 150-base pair paired-end reads. In the preliminary stages of the subsequent analysis, we conducted an initial filtering process to remove reads that contained adapters, poly-N sequences, and low-quality reads. The processed, or purified, reads were subsequently aligned to the reference genome using Bowtie2. For those reads that were not mapped, a backsplice algorithm was utilized to pinpoint junctions, and circRNAs were identified using the circRNA Finder, accessible at https://github.com/bioxfu/circRNAFinder. In the final phase, the circRNAs were annotated and compiled using the circRNA Anno tool within circRNA Finder. These annotations were used as reference sequences. The differential expression of circRNAs was analyzed using the DESeq2 package, which is based on a test using the negative binomial distribution. The parameters for determining differentially expressed circRNAs included a minimum absolute log2 (fold change) of ≥1 and a maximum FDR of ≤0.05 following multiple testing analysis.

### Animal experiments

The Institutional Animal Care Committee of the Second Affiliated Hospital of Zhejiang University granted approval for all the protocols employed in the animal research studies. Zmpste24^+/−^ mice were described previously [55]. The zmpste^+/−^ mice were provided by Gempharmatech Company. Zmpste24^+/−^ C57BL/6J mice were crossed to generate Zmpste24^−/−^ pups. For analysis, littermate controls were employed, and routine genotyping of all mice was performed using polymerase chain reaction (PCR) with DNA extracted from mouse tail samples. Biological analysis of vertebral intervertebral discs was conducted on mice aged 1 to 3 months, excluding neonatal animals. Mice in the experimental group received injections of the SDTET nanogel, DTET, or siRNA linker, and their intervertebral discs were examined at 3 months of age.

### DCFH-DA analysis

The 2,7-dichlorofluorescin diacetate probe (DCFH-DA) was used to assess cellular ROS levels. First, DCFH-DA was diluted in 0.01 M phosphate buffered saline (PBS) to the working concentration (20 nM), and the working concentration of DCFH-DA was added to hNPCs subjected to different treatments. The cells were incubated at 37°C for approximately 30 min. The solution was inverted and mixed every 3 to 5 min to ensure thorough contact between the probe and the cells. Finally, the cells were washed with PBS to remove excess DCFH-DA. The obtained cells were then observed under a fluorescence microscope.

### SA-β-gal staining

The SA-β-Gal staining procedure was carried out as previously described [56]. To summarize, we first washed the cells with PBS and then fixed them in a solution comprising 2% paraformaldehyde (PFA) and 0.2% glutaraldehyde for 5 min. After the fixation step, the cells underwent additional rinsing and were subsequently immersed in an SA-β-Gal solution (Beyotime) at 37°C for 12 h. Next, the hNPCs were cleansed twice with PBS and subsequently with methanol, after which they were left to dry naturally. The air-dried cells were then inspected using a Nikon Eclipse Ni-U microscope. In each culture dish, we quantified both the total cell count and the total number of cells exhibiting positive SA-β-Gal staining in 3 randomly chosen areas.

### Immunofluorescence

The hNPCs were fixed with 4% paraformaldehyde, and the nucleus pulposus sections were subjected to deparaffinization and retrieval of tissue antigens. Both the cell types and nucleus pulposus were then permeabilized with 0.3% Triton X-100 for 15 min, followed by a blocking phase with 1% BSA at 37°C for 60 min. After incubating overnight with primary antibodies, the cells were incubated with Alexa Fluor-conjugated secondary antibodies (Thermo Fisher Scientific), followed by another incubation period of 1 h at 37°C. Subsequently, the cell nuclei were labeled with DAPI (Cell Signaling Technology) for 5 min at ambient temperature. For visualization, a Nikon confocal microscope system was used. Comprehensive information regarding the antibodies employed in this study can be found in Table S4.

### Western blots

We extracted whole-cell lysates from tissues or cells utilizing a protein extraction kit (Beyotime). NPCs were lysed using radio-immunoprecipitation assay (RIPA) buffer (Beyotime) supplemented with 1% phenylmethylsulfonyl fluoride (PMSF) (Sigma). A bicinchoninic acid (BCA) assay kit (Vazyme) was used to quantify the protein concentrations. Subsequently, the proteins were separated via SDS-PAGE, transferred onto polyvinylidene fluoride (PVDF) membranes, and blocked with 5% skim milk for 2 h. The membranes were subsequently incubated overnight at 4°C with primary antibodies. After TBST washes, the membranes were exposed to secondary antibodies for 1 h, and chemiluminescent substrates were used for detection. A comprehensive list of all the antibodies used in this study can be found in Table S4. The blot images were captured using a Tanon 3500 system, and ImageJ (v1.8.0) was used to quantify the gray values.

### sGAG assay

hNPCs maintained in culture were subjected to lentivirus transfection (multiplicity of infection, MOI = 10) and subsequently incubated for a period of 24 h. Posttransfection, the cells were cultivated in fresh DMEM supplemented with 1% antibiotics devoid of FBS for 3 days. During this interval, we collected the supernatant from the cell culture to assess the sGAG content. In siRNA-based experiments, hNPCs were transfected with siRNA utilizing the SDTET nanogel at a concentration of 40 nM for 24 h, followed by analysis of the sGAG content in the culture supernatant. The quantification of sGAG in the supernatant was performed using the DMMB dye assay, where the absorbance was measured at 525 nm, and the results were normalized against those of the MTT (Sigma Aldrich) assay.

### Total RNA isolation and real-time RT-qPCR

RNA was extracted using TRIzol reagent (TaKaRa Bio). Subsequently, RT-qPCR analysis was carried out using the StepOnePlus Real-time PCR System (Applied Biosystems, CA) and the SYBR Premix Ex TaqTM Kit (TaKaRa Bio). As a reference gene for normalization, 18S rRNA was chosen for normalization. The results were calculated using the 2^–ΔΔCt^ method. All primers used in this study were provided by Sangon Biotech (Shanghai, China), and their specific details can be found in Table S3.

### Lentiviral construction and cell transfection

To construct the lentivirus, the circRNA hsa_circ_0007909 (renamed circATXN1) was synthesized by the Hanbio Company (Shanghai, China). First, primers for circATXN1 were designed, and after PCR amplification, the primers were inserted into pHBLV (Table S3). The sequence integrity was confirmed through Sanger sequencing. The plasmid without the insertion of circATXN1 was used as the negative control group. All these vectors were ultimately cloned and inserted into lentiviruses. Transfection of young hNPCs was performed using lentiviruses. Transfection of aged hNPCs was performed using the SDTET nanogel for siRNA. The sequences of the siRNAs used in this investigation are documented in Table S2.

### RNase R digestion

Total RNA was subjected to 2 distinct procedures: one portion was treated with RNase R (4 U/μg) (Beyotime) and incubated for 20 min. The other portion was kept as a control and was not subjected to any treatment. After these treatments, the processes of reverse transcription and RT-qPCR were executed following the procedures outlined in the RNA extraction and RT-qPCR section.

### CCK-8 analysis

The cytotoxic effects of the liposomes and SDTET nanogels on hNPCs were evaluated using the CCK-8 assay (Vazyme). We seeded the cells in 96-well plates at a density of 5 × 10^3^ cells/100 μl. Each sample was tested with 3 replicates. After adding 10 μl of CCK-8 solution, the absorbance at 450 nm was measured using an automated microplate reader (BioTek) at the 0- and 48-h time points.

### RNA fish

We used a fluorescent in situ hybridization kit (Thermo) to investigate the subcellular distribution of circATXN1. Digoxigenin (DIG)-labeled circATXN1 probes were used for hybridization. Subsequently, the samples were examined using a confocal microscope (Nikon). The sequence used for RNA in situ hybridization was as follows: DIG-labeled circATXN1 probe: 5′DIG-ACGAUGCUCCUGUACCA-DIG3′.

### Methylated RNA immunoprecipitation assay

We performed methylated RNA immunoprecipitation (MeRIP) using a MeRIP kit (Bersin Bio). Initially, total RNA was extracted, and 10 μl of each RNA sample was retained as an input sample and stored at −80°C. For the immunoprecipitation (IP) step, an m6A-specific antibody, 200 μl of 1× IP reagent, and 2 μl of RNase inhibitor were added to the remaining RNA samples. This mixture was incubated in a vertical mixer at 4°C.

Protein A/G beads were prepared by washing 30 μl of beads for each MeRIP sample twice with 1× IP reagent. Subsequently, the sections were blocked with 1× IP reagent containing 0.5 mg/ml BSA at 4°C. The beads that had been blocked were subsequently rinsed twice with 100 μl of 1× IP reagent. Protein A/G bead binding to the antibody was achieved by incubating the prepared beads with the sample–antibody hybrid solution. Afterward, the sections were washed 3 times with 1× IP reagent and incubated with 1× IP reagent at 50°C for 45 min, after which the mixture was mixed every 10 min. RNAs were extracted and subsequently subjected to reverse transcription, PCR, and qPCR experiments. The primers used to target the specific m^6^A sites for the following experiments are provided in Table S3.

### RNA-binding protein immunoprecipitation

RIP assays were performed utilizing the RIP RNA-Binding Protein Immunoprecipitation Kit (Promega Bio). We collected approximately 1×10^5^ cells and suspended them in 100 μl of RIP lysis buffer containing equal volumes of protease and RNase inhibitors. The cell lysates were then subjected to overnight incubation at 4°C with either 5 mg of control mouse immunoglobulin G (IgG) or antibody-coated beads with continuous rotation. After the incubation period, proteinase K treatment was performed, and the immunoprecipitated RNAs were extracted using phenol–chloroform extraction.

### RAP assay

The RAP procedure was carried out employing an RAP kit (Bersin Bio). The key steps in this experiment included cell cross-linking, cell pellet collection, homogenization, DNA removal, probe preparation, bead preparation, hybridization and capture, protein elution, protein precipitation, protein dissolution, and quality control analysis. The proteins obtained were subsequently utilized for Western blotting or MS. The resulting supernatants were transferred to a loading tube for MS identification. Mass spectrometric analysis was outsourced to Hangzhou Precision Medicine Research Center (Hangzhou, China).

### RNA pull-down assay and MS analysis

RNA pull-down experiments were conducted employing an RNA–Protein Pull-Down Kit (Bersin bio). In brief, cell lysates were prepared through ultrasonication in RIP buffer. These lysates were initially cleared by preincubation with streptavidin magnetic beads. Biotinylated RNA synthesized in vitro was bound to streptavidin-coated magnetic beads and mixed with the cell lysates at 4°C for a period of 4 h. Proteins were then extracted with elution buffer. The mass spectrometric analysis was then performed at the Hangzhou Precision Medicine Research Center (Hangzhou, China).

### Assembly of the siRNA-embedded DNA nanogel and DOTAP liposomes

The ssDNAs Tetra A, Tetra B, Tetra C, and Tetra D used for the assembly of DTETs are shown in Table S2. DET was first obtained by mixing these 4 strands together at an equimolar ratio (5 μM) in 1× TAE/Mg^2+^ buffer and then undergoing a rapid cooling process. To further obtain the siRNA-loaded DTET-nanogel, sticky ends complementary to the terminal extension of DTETs were introduced at the 5′ terminus of the siRNA linker. The assembled DTETs (3 μM) were incubated with the siRNA linker (20 μM) in 1× TAE/Mg^2+^ buffer at an optimized molar ratio (DTETs/siRNA linker) of 1:1.8 at 25°C for 1 h. The DOTAP liposomes were prepared by the conventional evaporation method [57].

### Native PAGE analysis

PAGE, a straightforward electrophoretic technique, is employed to assess biological macromolecules, such as nucleic acids, based on their hydrodynamic dimensions and electric charge. The construction of the DTET and SDTET nanogels was verified via PAGE (10%). The gels were run in 1× TBE buffer at 450 V and 4°C. After electrophoresis, the gels were immersed in Gel-Red solution for staining, and the images were visualized using a ChampGel 7000 (Sagecreation, China).

### Agarose gel electrophoresis

The samples were analyzed by 3% agarose gel electrophoresis at 4°C (100 V) for approximately 45 min in 1 × TBE buffer. The bands were stained with GelRed and visualized with a ChampGel 7000 (Sagecreation, China).

### DLS analysis and AFM

The size distribution and zeta potential of the DTET and SDTET nanogels were assessed using DLS measurements conducted on a Zetasizer Nano ZS instrument (Malvern Instruments Ltd.). AFM images were obtained using a MultiMode 8 AFM in ScanAsyst-Air mode.

### Histological evaluation

At the specified time intervals, the zmpste24^−/−^ mice were euthanized, and their nucleus pulposus was collected and preserved in 10% formalin for subsequent histological assessment. The specimens were dehydrated through a series of alcohol gradients and subsequently embedded in paraffin. The resulting sections of the intervertebral disc were 5 μm thick. The staining procedures included SO-fast green or HE staining. Two blinded observers assessed cellularity and morphology using a pre-established grading scale [58].

### Micro-CT scanning

The intervertebral disc was soaked in iohexol solution (320 mg I/ml) for 24 h in preparation for discography. Subsequently, a micro-CT examination was conducted (Skyscan1276, Bruker). The purpose of this examination was to assess the morphology of the nucleus pulposus and changes in intervertebral disc height and to generate a 3-dimensional image of the disc. Subsequent Piffmann classification assessments were also conducted. The scanning parameters employed were as follows: 70 kV, 200 mA, and an average slice thickness of 10 μm.

### Quantification and statistical analysis

The data are presented as the mean ± SD. The statistical significance of the differences between groups was determined through one-way analysis of variance followed by Tukey’s post hoc test or a nonparametric test. Each experiment was repeated a minimum of 3 times, yielding consistent outcomes. All the statistical analyses were performed using SPSS software version 22.0 (SPSS, Inc., Chicago, USA), and *P* < 0.05 indicated statistical significance.

## Data Availability

The data for bioinformatics analysis in this study are submitted to GEO databases. Other data and materials from other public databases or biotechnical company are provided in our paper or Supplementary Materials.
