# Supplementary material for: Silencing circATXN1 in Aging Nucleus Pulposus Cell Alleviates Intervertebral Disc Degeneration via Correcting Progerin Mislocalization
Source: Research (Wash D C). 2024 Mar 23;7:0336. doi: 10.34133/research.0336 (PMC10964222; doi:10.34133/research.0336)
Supplement: Supplementary 1 — Tables S1 to S4 Figs. S1 to S7 [file research.0336.f1.docx]

**Supporting information**

**Table S1. Descriptive characteristics of human patients with IVDD. BMI: Body mass index.**

| Patient ID | Gender | Age(years) | Height(cm) | Weight(kg) | BMI(kg/m^2^) |
| --- | --- | --- | --- | --- | --- |
| ZR-013 | F | 73 | 158.2 | 61.3 | 24.5 |
| ZR-026 | F | 75 | 157.3 | 55.6 | 22.5 |
| ZR-031 | F | 72 | 161.3 | 57.7 | 22.2 |
| ZR-045 | F | 77 | 159.8 | 66.1 | 25.9 |
| ZR-052 | F | 70 | 163.5 | 59.2 | 22.1 |
| ZR-061 | F | 13 | 148.6 | 53.0 | 24.0 |
| ZR-078 | F | 12 | 149.7 | 49.2 | 22.0 |
| ZR-082 | F | 13 | 150.5 | 48.9 | 21.6 |
| ZR-089 | F | 14 | 147.1 | 51.2 | 23.7 |
| ZR-095 | F | 12 | 146.6 | 47.9 | 22.3 |

**Table S2. The sequences used for construction of DTET, siRNA linker and SDTET nanogel.**

| **Name** | **sequence (5’to3’)** |
| --- | --- |
| **Tetra A** | **TTG ACC TGT GAA TT ACA TTC CTA AGT CTG AAA AT TAC AGC TTG CTA CAC G AAG AGC CGC CAT AGT A** |
| **Tetra B** | **TTG ACC TGT GAA TT TAT CAC CAG GCA GTT GAC AGT GTA GCA AGC TGT AAT AGA TGC GAG GGT CCA ATA C** |
| **Tetra C** | **TTG ACC TGT GAA TT TCA ACT GCC TGG TGA TAA AAC GAC ACT ACG TGG GAA TCT ACT ATG GCG GCT CTT C** |
| **Tetra D** | **TTG ACC TGT GAA TT TTC AGA CTT AGG AAT GTG CTT CCC ACG TAG TGT CGT TTG TAT TGG ACC CTC GCA T** |
| **Sense linker** | **rUrUrCrArCrArGrGrUrCrArArGrUrArCrArGrGrArGrCrArUrCrGrUrGrCrArU** |
| **Antisense linker** | **rUrUrCrArCrArGrGrUrCrArArArUrGrCrArCrGrArUrGrCrUrCrCrUrGrUrArC** |
| **Cy5-Antisense linker** | **Cy5-rUrUrCrArCrArGrGrUrCrArArArUrGrCrArCrGrArUrGrCrUrCrCrUrGrUrArC** |
| **ms-Sense linker** | **rUrUrCrArCrArGrGrUrCrArArUrCrCrUrArUrGrArArGrArCrArUrGrGrUrArA** |
| **ms-Antisense linker** | **rUrUrCrArCrArGrGrUrCrArArUrUrArCrCrArUrGrUrCrUrUrCrArUrArGrGrA** |
| **has-siRNA** | **rArUrGrCrArCrGrArUrGrCrUrCrCrUrGrUrArC** |
| **ms-siRNA** | **rUrUrArCrCrArUrGrUrCrUrUrCrArUrArGrGrA** |

Notes:

1. Red sequences are sticky ends for hybridization to form SDTET-nanogel with siRNAs embedded;

2. The Cy5 labeled antisense linkers were used for cellular uptake study.

3. All DNA and RNA sequences were ordered from Sangon Biotech.

**Table S3. List of PCR primers.**

| Gene name | Primer sequence（Forward） | Primer sequence（Reverse） |
| --- | --- | --- |
| hsa-Cdkn1a | TGTCCGTCAGAACCCATGC | AAAGTCGAAGTTCCATCGCTC |
| hsa-Cdkn2a | GATCCAGGTGGGTAGAAGGTC | CCCCTGCAAACTTCGTCCT |
| hsa-ATXN1 | TCGGTGGAGCTTGGTTTACAA | GGGAGGACCCAATGAACTGG |
| hsa-circATXN1 | GCACATGGTACAGGAGCATCGT | TGTAGTGGCAGTGGAGGAGGAG |
| mmu-circATXN1 | GGAAGGAAGTGGCTGCTTGTGG | TGTGGAGGGAGTGGAGGAAGAG |
| hsa-18s | CGCCGCTAGAGGTGAAATTC | TTGGCAAATGCTTTCGCTC |
| mmu-β-Actin | GGCTGTATTCCCCTCCATCG | CCAGTTGGTAACAATGCCATGT |
| m^6^A primer 1 | ACAACAGAATGCCGAATGTG | GTGCAGGAGAGGAAATCCAA |
| m^6^A primer 2 | GAGAAACCAGGGGAGACAGA | CGGCTGTCAACAATCACAAC |
| m^6^A primer 3 | CCAGAGCCCTGTTGACTCAC | CCAAGAAAGCCCTCTTATTCC |
| m^6^A primer 4 | AAAATCTCAGCGGTGTGAGG | ACATCCTTGCCCATTGTACC |
| m^6^A primer 5 | CTCCCAAAGTGCTGGGATTA | CACACATGACTGCCTGCTCT |
| m^6^A primer 6 | TTTCAGACCCGTAACTTCTGC | GCTGCACGTCTCTACACAGC |
| m^6^A primer 7 | TCTCGGATTTCTAGCCTCCA | TGTTAGTTTCCTGGGGTTGC |
| m^6^A primer 8 | AATCGGAGACCTGGAGCAT | AAATTCCAGTCCTGGGTGTG |
| m^6^A primer 9 | AACCAGGTCACCAGAGGTTG | CCAGCTTGAGCGTCTCTTTC |
| LV-hsa_circ_0007909-E/B | GACATTAATATTTCTTCTTTCGA  ATTCTAATACTTTCAGGAGCATCGT | GTATGGAGTTGTTAGCTAGGATC  CAGTTGTTCTTACCTGTACCATGT |

**Table S4 Antibody Name and Source**

| Antibody | Product number |
| --- | --- |
| P16INK4a | 18769 (Cell Signaling Technology) |
| P21 | 2947 (Cell Signaling Technology) |
| SOX9 | sc-166505 (Santa Cruz) |
| MMP13 | sc-515284 (Santa Cruz) |
| HNRNPA2B1 | sc-32316 (Santa Cruz) |
| COL2 | sc-52658 (Santa Cruz) |
| Progerin | 05-1231 (Sigma) |
| IGF-1Rβ | sc-81464 (Santa Cruz) |
| ACAN | AB1031 (Sigma) |
| Lamin A/C | sc-376248 (Santa Cruz) |


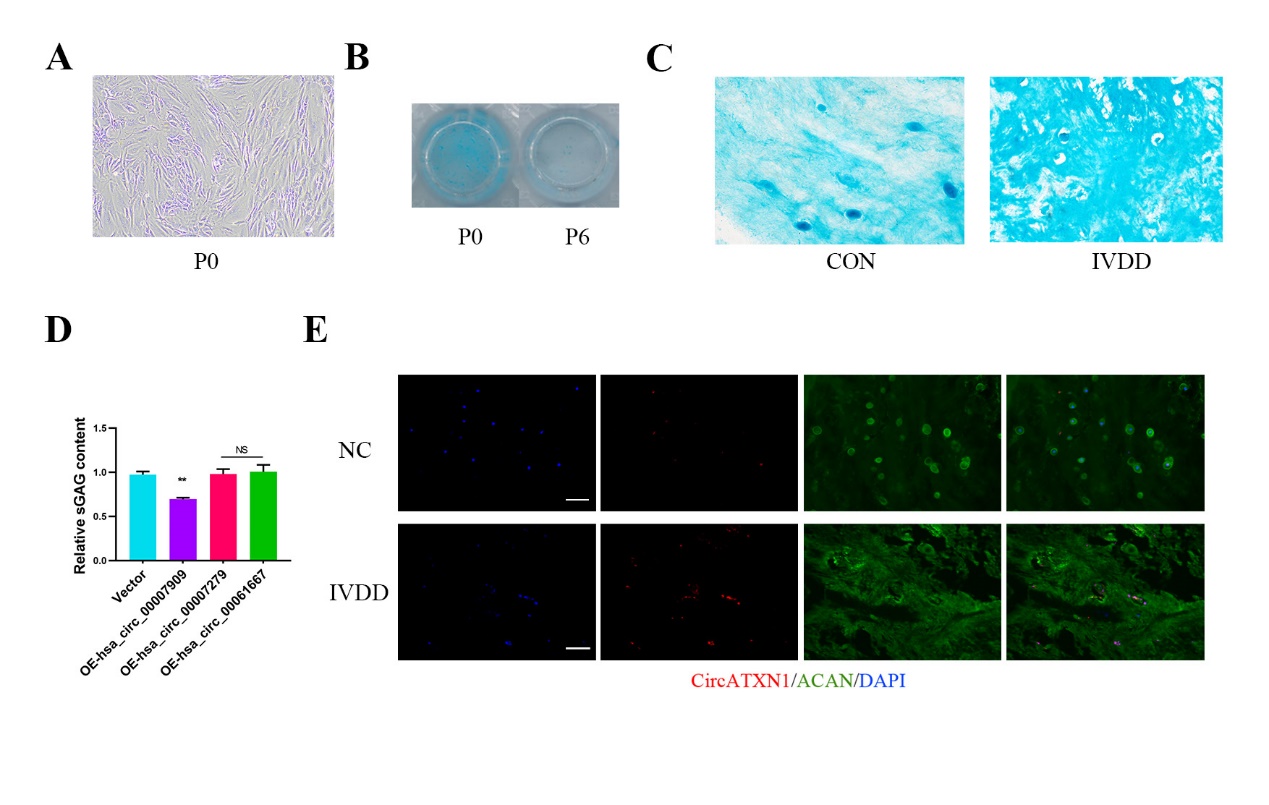


**fig. S1 Aging-related phenotypes appear in degenerated nucleus pulposus tissue/cell samples.**

(A) Toluidine Blue O staining of primary hNPCs. (B and C) Alcian blue staining of hNPCs and nucleus pulposus tissues. (D) Following lentiviral overexpression of hsa_circ_0007909, hsa_circ_0007279, and hsa_circ_0061667, sGAG content is measured using the DMMB method in young hNPCs. (E) RNA in situ hybridization against circATXN1 and immunofluorescence staining of and ACAN in the nucleus pulposus tissues. Scale bar, 100 μm. NC: normal nucleus pulposus tissue; IVDD: degenerated nucleus pulposus tissue.


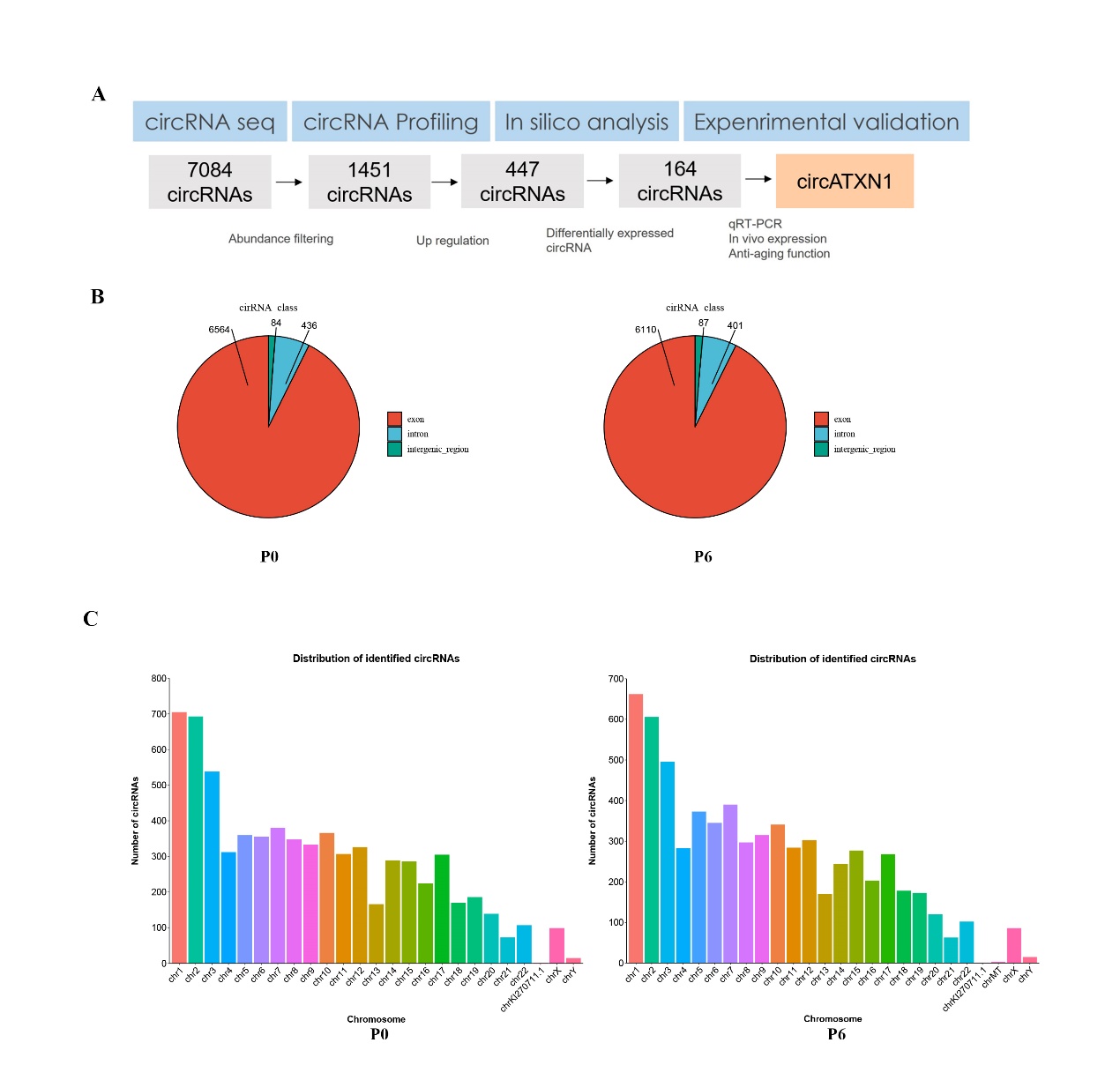


**fig. S2 Analysis of the composition and origin of circRNAs obtained from gene sequencing.**

(A) The procedure of Circular RNA sequencing. (B) The composition types of circRNAs in P0 and P6 hNPCs. (C) The chromosomal distribution of circRNAs in P0 and P6 hNPCs.


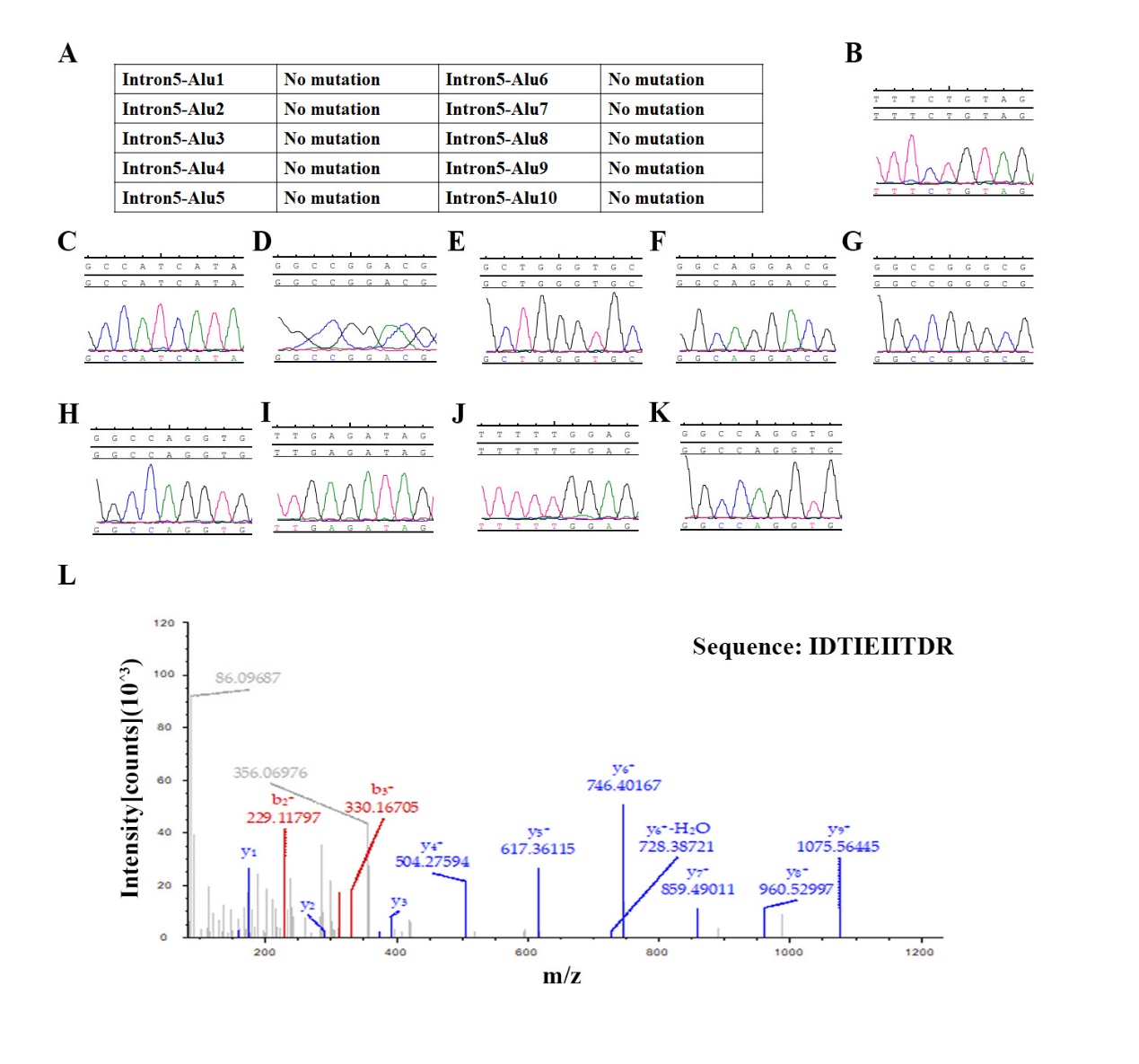


**fig. S3 Splicing regulation of Pre-ATXN1.**

(A) Table summarizing the mutated *Alu* site positions. (B-K) Sanger sequencing of the *Alu* sites within intron 5.

(L) Mass spectrogram of HNRNPA2B1 protein.


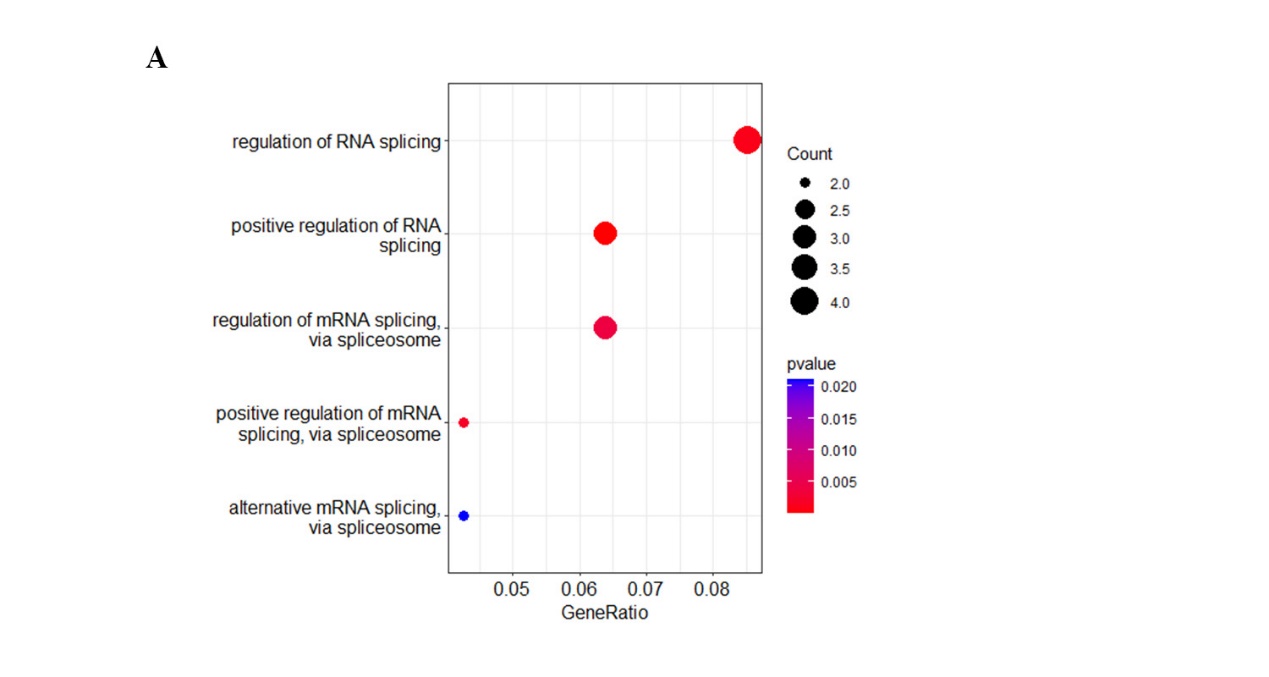


**fig. S4 GO analysis of the mass spectrometry results from RAP-purified proteins.**

(A)Bubble chart depicting the results of the GO analysis.


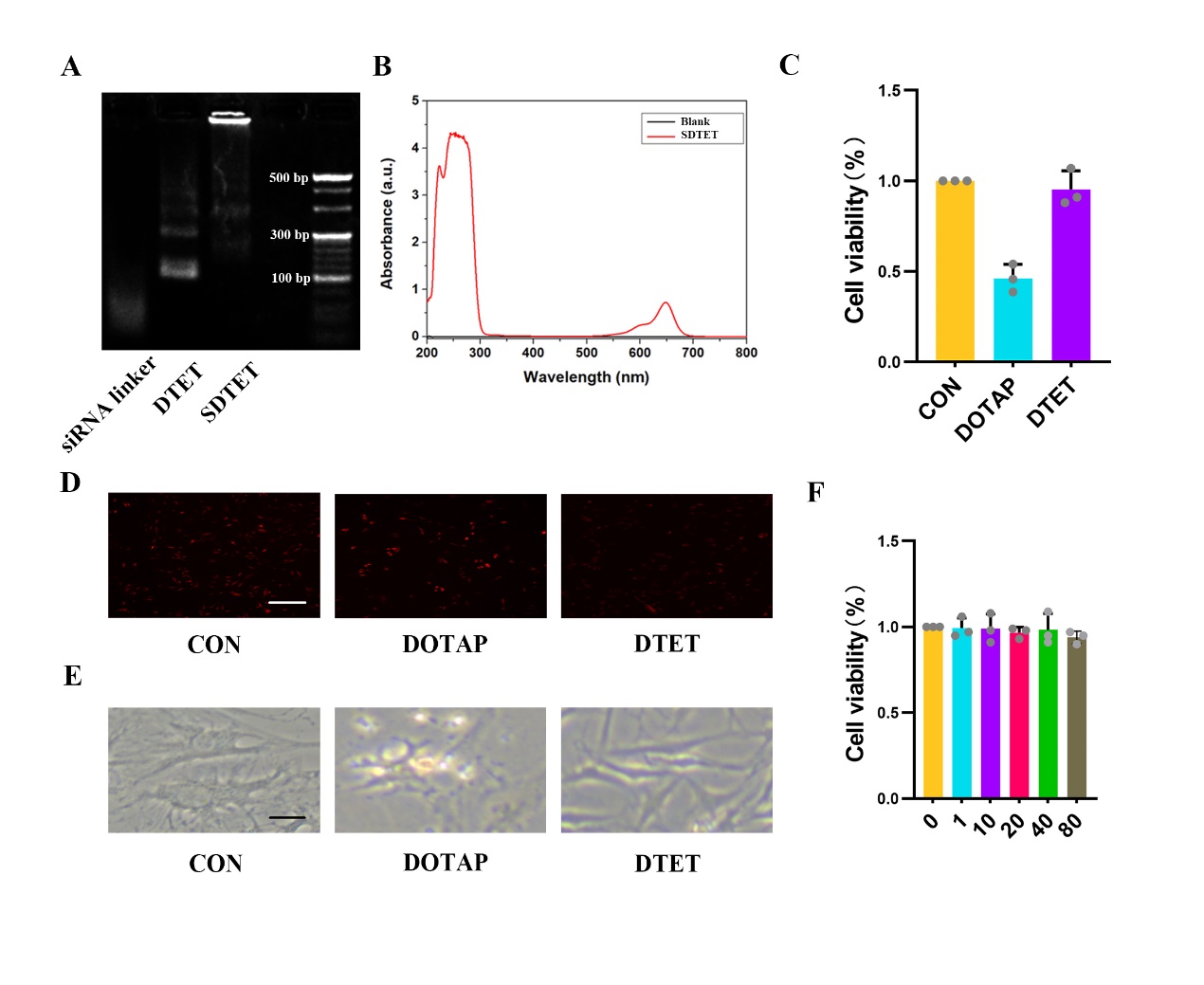


**fig S5. Characterizations of SDTET.**

(A) Agarose gel electrophoresis analyze. (B) Ultra-violet absorption spectra of Cy5-SDTET. (C) Cell viability assay using CCK-8 for assessing DOTAP liposome (25 μg/ml) and SDTET (25 μg/ml) cytotoxicity. (D) Immunofluorescence staining of DCFH in aging hNPCs treated with DOTAP liposome (25 μg/ml) and DTET (25 μg/ml) for 2 hours in vitro. Scale bar, 100 μm. (E) The morphological change of the aging hNPCs treated with with DOTAP liposome (25 μg/ml) and DTET (25 μg/ml) for 2 hours in vitro. Cells were subjected to inverted microscope observation. Scale bar, 20 μm. (F) Cell viability assay using CCK-8 for assessing SDTET cytotoxicity at different concentrations (nM).


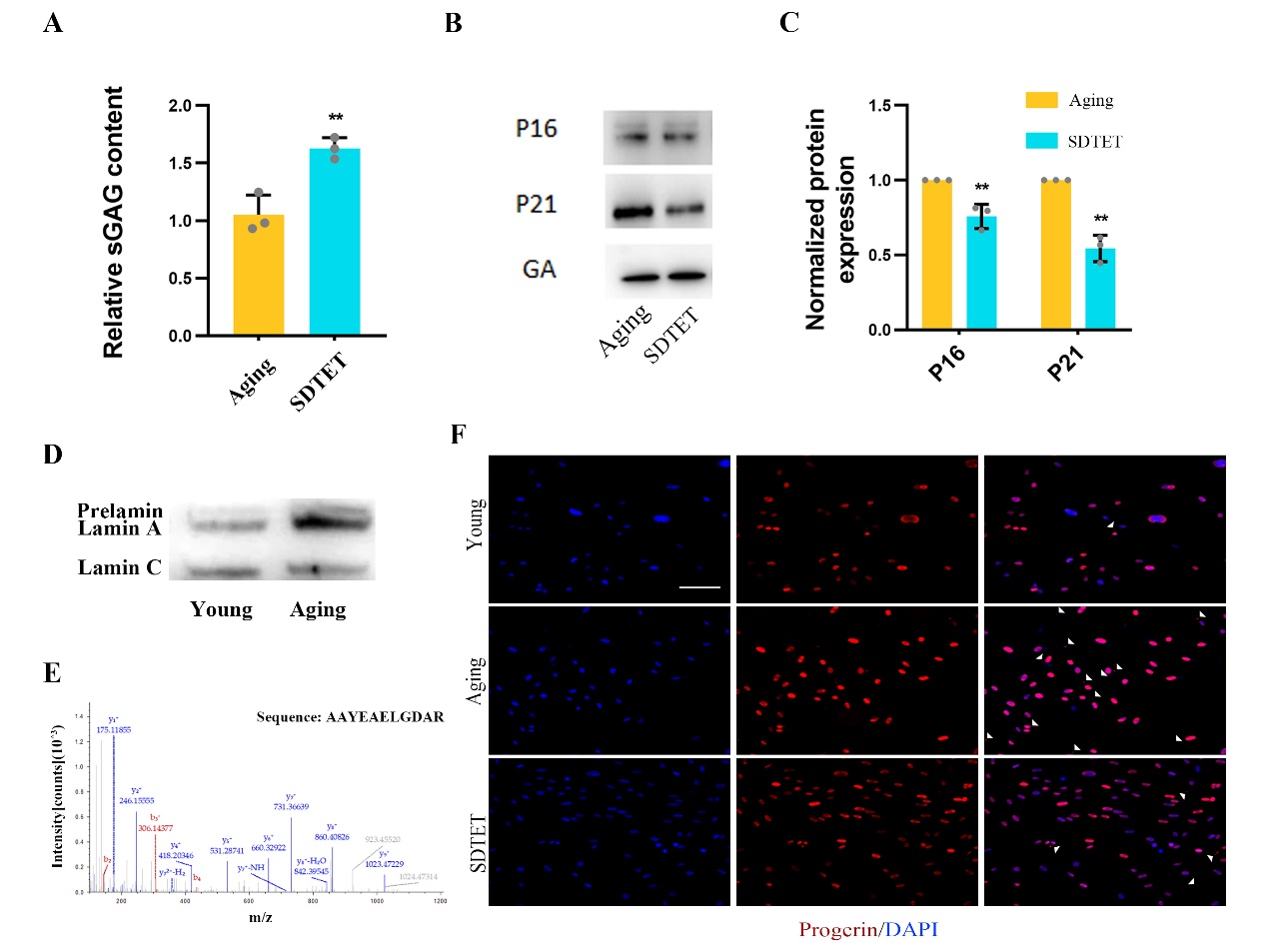


**fig. S6. circATXN1 regulates the aging and cellular remodeling phenotype of hNPCs.**

(A) DMMB analysis of cellular sGAG content. (B-C) Western blot analysis of P16 and P21 protein expression.NC, P6 hNPCs group. (D) Western blot analysis shows there is an accumulation of prelamin in aging NPCs. (E) Mass spectrogram of Progerin protein. (F) Immunofluorescence demonstrates hNPCs with cytoplasmic mislocalization. The white arrows indicate mislocalized cells.


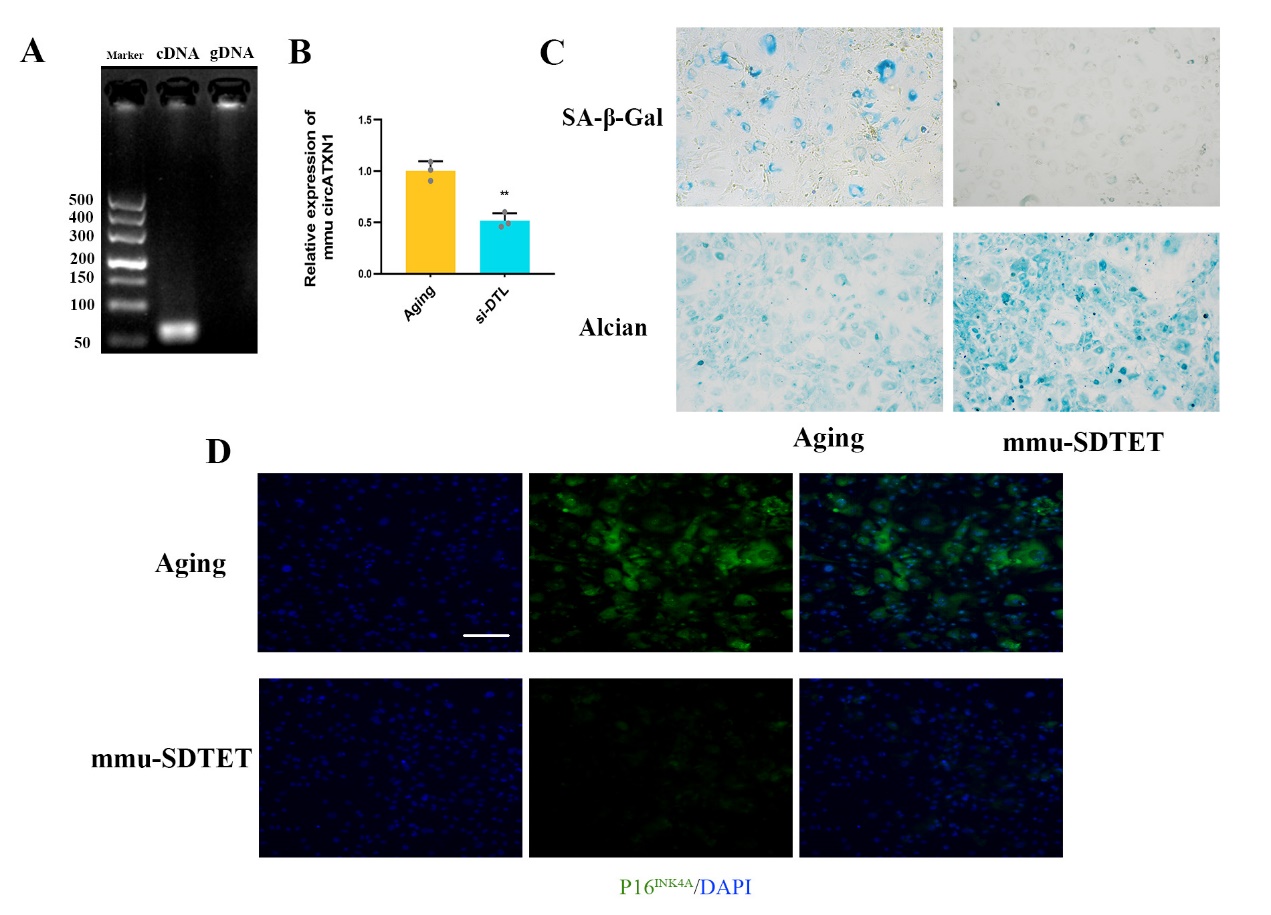


**fig S7. circATXN1 regulates the aging and cellular remodeling phenotype of mouse NPCs.**

(A) ms-circATXN1, along with 18s, were amplified from cDNA or gDNA from aging mNPCs by PCR assays, respectively. (B) RT-qPCR analysis of ms-circATXN1 levels in aging mNPCs with and without SDTET treatment (n = 3 biological replicates). (C) SA-β-Gal and Alcian staining of mNPCs with and without SDTET treatment. (D) Immunofluorescence staining of p16^INK4a^ in aging mNPCs with and without SDTET treatment. Scale bar, 100 μm.
